# PIEZO1 mediates mechanical reprogramming of neutrophils for proangiogenic specialization in the lung

**DOI:** 10.1172/JCI183796

**Published:** 2025-06-02

**Authors:** Jin Wang, Wenying Zhao, Wenjuan Bai, Dong Dong, Hui Wang, Xin Qi, Ajitha Thanabalasuriar, Youqiong Ye, Tian-le Xu, Hecheng Li, Paul Kubes, Bin Li, Jing Wang

**Affiliations:** 1 Shanghai Institute of Immunology,; 2 Department of Thoracic Surgery, Ruijin Hospital, and; 3 Department of Anatomy and Physiology, Shanghai Jiao Tong University School of Medicine, Shanghai, China.; 4 Department of Pharmacology and Therapeutics, McGill University, Montreal, Quebec, Canada.; 5 Snyder Institute for Chronic Diseases, Cumming School of Medicine, University of Calgary, Calgary, Alberta, Canada.

**Keywords:** Immunology, Pulmonology, Vascular biology, Ion channels, Microcirculation, Neutrophils

## Abstract

Neutrophils are the most abundant immune cells that constantly patrol or marginate inside vascular beds to support immune homeostasis. The extent to which neutrophils undergo reprogramming in response to the changes in vascular architecture and the resultant biological implications of such adaptations remain unclear. Here, we performed intravital imaging and transcriptional profiling to investigate neutrophil behavior across different tissues. Our findings revealed that neutrophils had significant deformability and spontaneous calcium signaling while navigating through the narrow pulmonary vessels. Pulmonary neutrophils exhibited unique transcriptional profiles and were specialized for proangiogenic functions. We found that the mechanosensitive ion channel Piezo-type mechanosensitive ion channel component 1 (PIEZO1) was essential for neutrophil reprogramming. Deletion of *Piezo1* in neutrophils ablated the lung-specific proangiogenic transcriptional signature and impaired capillary angiogenesis in both physiological and pathological conditions. Collectively, these data show that mechanical adaptation of neutrophils within the pulmonary vasculature drives their reprogramming in the lungs and promotes pulmonary vascular homeostasis.

## Abstract 2

Neutrophil fate and function driven by deformation

## Graphical abstract

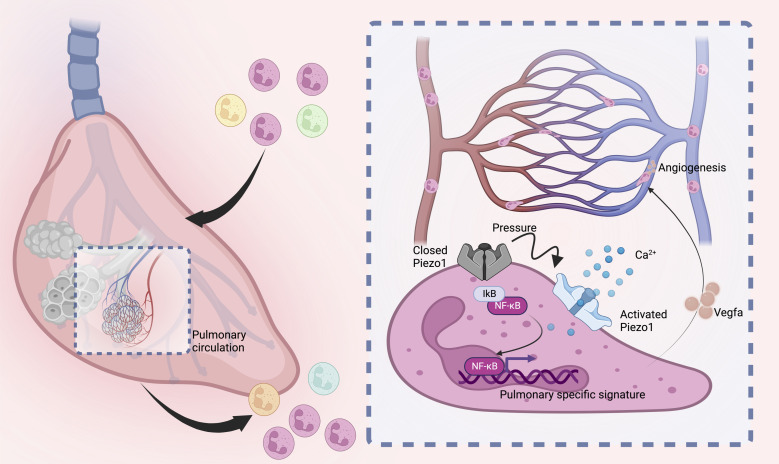

## Introduction

Neutrophils constitute a significant proportion of circulating leukocytes in both humans and mice, accounting for approximately 50%–70% and 10%–25% of the total leukocyte count, respectively. Known as terminally differentiated and short-lived cells in peripheral blood (PB), neutrophils have been suggested to have limited plasticity and heterogeneity. However, a growing body of research using deep profiling techniques has suggested that distinct neutrophil subpopulations exist in steady state, infectious and inflammatory diseases, tissue repair, and cancer (1). The drivers that generate these neutrophil subpopulations have not been well characterized.

Many tissue-resident immune cells, including macrophages and innate lymphoid cells, reside in distinct niches across organs, contributing to tissue homeostasis and the timely response to local microenvironment perturbations (2). Traditionally, neutrophils are not defined as tissue-resident cells because they continuously circulate in the blood. Nevertheless, under homeostasis, neutrophils can be found in direct contact with endothelial cells (ECs) of certain vascular beds and are retained in the blood vessels of certain tissues for a prolonged time. This population of neutrophils is called the “marginated pool.” The tissues that contain these marginated pools are the lung, liver, and spleen (3). It has been documented that neutrophils can adapt to the tissue environment they infiltrate and acquire specialized phenotypes and functions (4). However, whether and how marginated neutrophils receive “input” from the tissue microenvironment and the physiological environment cues that shape neutrophil phenotype and functions are not well studied.

The tissue microenvironment consists of various biological, chemical, and physical components that are favorable for optimal organ function. Soluble factors, xenobiotic factors, nutrients, and metabolites have long been recognized as essential environmental cues of the local cellular microniches. Conversely, cells are equally sensitive to some physical properties of the environment, including tissue stiffness, physical confinement, and mechanical tension (5). Several studies have revealed mechanisms by which cells perceive mechanical cues from the external microenvironment and transduce them into molecular signals and genetic regulations to coordinate the cellular response (6). Neutrophils display considerable plasticity in shape and migratory behaviors while patrolling across different organs and experiencing different mechanical environments. However, the contribution of mechanical sensing in regulating neutrophil phenotype and functions remains unclear.

Here, we performed intravital imaging and integrative behavior analysis of neutrophils from multiple tissues in physiological states. Through this analysis, we observed unique migratory behavior and intracellular signaling in pulmonary neutrophils. Further analysis revealed that mechanical sensing via the mechanically sensitive ion channel Piezo-type mechanosensitive ion channel component 1 (PIEZO1) drove the phenotypical and functional specialization of pulmonary neutrophils. Through intravital imaging and in vitro microfluidic mimetics, we demonstrated that vascular confinement triggered PIEZO1 activation, leading to the expression of proangiogenic signatures in neutrophils. Moreover, we revealed that neutrophil deficiency of *Piezo1* led to the failure of mounting an effective antibacterial response and resulted in decreased pulmonary angiogenesis in both physiological and pathological conditions. Consequently, we have discovered a process of tissue adaptation and specialization for pulmonary neutrophils that is driven by PIEZO1 and is crucial for maintaining vascular homeostasis.

## Results

### Neutrophil behavioral landscapes across different tissues.

**Figure 1:**
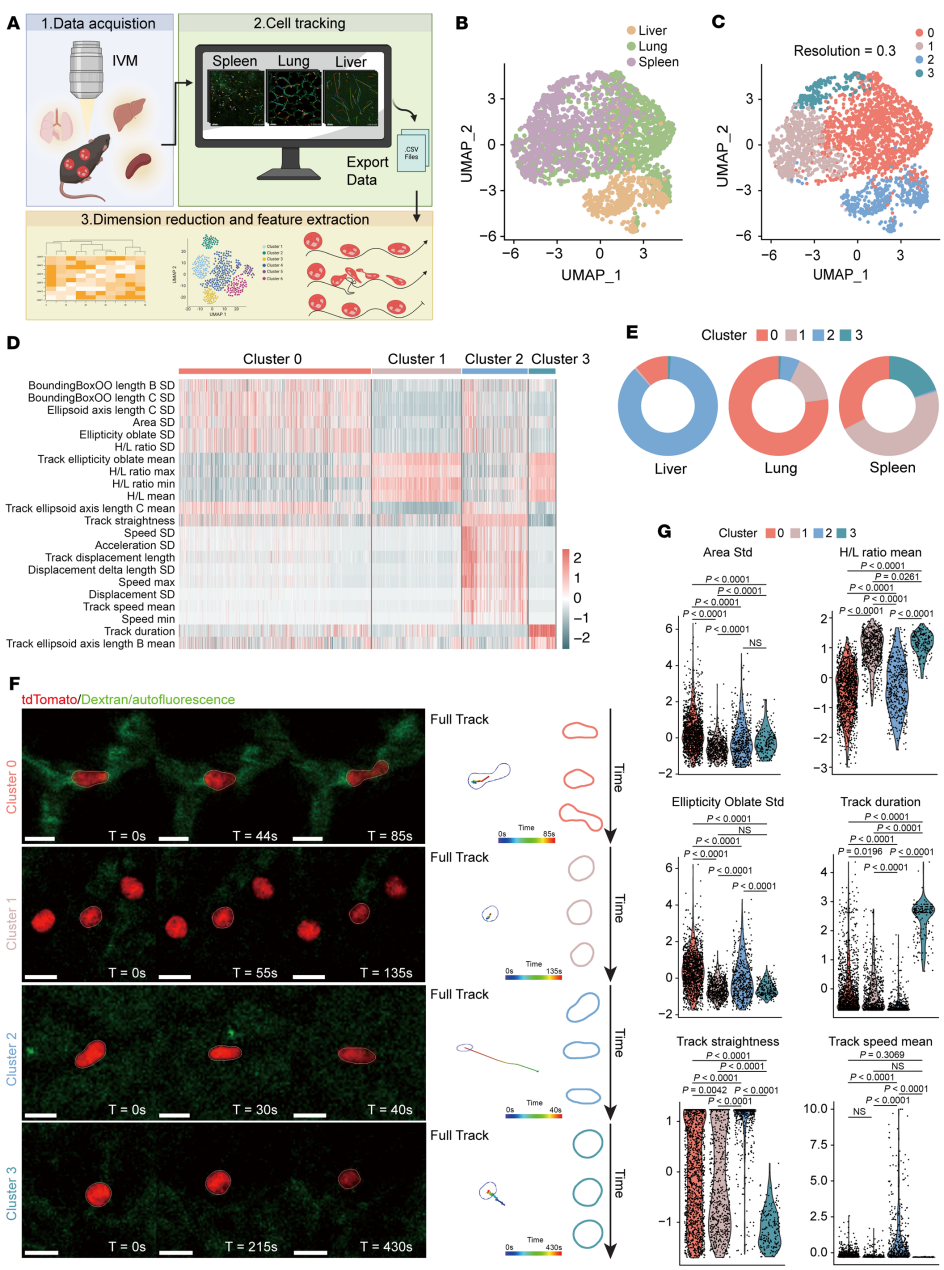
Neutrophil behavior landscapes in different tissues. (**A**) Schematic of experiment workflow. Parameters describing the motion and shape of hundreds of cells were extracted from intravital microscopy (IVM) imaging data analysis. A nonlinear reduction technique (UMAP) was performed to visualize the data in a low-dimensional space. (**B** and **C**) UMAP of behavioral traits of 2,500 neutrophils from liver, lung, and spleen. Each dot corresponds to a single cell, colored by sample origin (**B**) or cell type (**C**). (**D**) Heatmap showing the morphology and kinetics parameters for different neutrophil subsets. (**E**) Proportions of the 4 neutrophil clusters in 3 tissues. (**F**) Examples of neutrophil snapshots from intravital imaging for each cluster (left panel), their trajectory (middle panel), and their shapes (right panel). Scale bars: 15 μm. (**G**) Violin plots for the indicated parameters across the 4 behavioral groups; each dot corresponds to a single cell. Data were analyzed using a univariate multinomial model. Statistical significance was determined by 1-way ANOVA with Dunnett’s post hoc test.

Neutrophils experience varying physical conditions as they travel through the circulatory system. These include changes in vessel diameters, blood flow velocity, and vessel tortuosity. Here, we hypothesized that neutrophils behave differently in tissues with distinct vessel architectures, which may also influence their genetic and protein content. To verify our hypothesis, we performed intravital imaging with lymphocyte antigen 6G tdTomato–transgenic (*Ly6g^tdTomato^*-transgenic) mice, in which only neutrophils were labeled with tdTomato (Supplemental Figure 1, A and B; supplemental material available online with this article; https://doi.org/10.1172/JCI183796DS1). Subsequently, we imaged multiple tissues at a defined time interval and tracked in total approximately 2,500 neutrophils from lung, liver, and spleen (Supplemental Video 1). To gain more insight into the behavior and morphological adaptation of neutrophils during circulation in different tissues, we applied the recently introduced “behavior landscape” approach. This method integrates multiple parameters to comprehensively reflect cellular dynamics and morphology (7) (Figure 1A). A total of 38 parameters describing features of kinetics and morphology of hundreds of cells from each tissue were extracted for further analysis (Supplemental Table 1). Neutrophils imaged from different tissues were then projected into different clusters (Figure 1B). Clustering with uniform manifold approximation and projection (UMAP) revealed distinct subpopulations related to neutrophil dynamics and morphology (Figure 1, C and D). The proportion of these subsets in different organs was consistent between different mice, indicating the conserved nature of these behavioral traits (Figure 1E and Supplemental Figure 1C). The major behavioral traits in each tissue were different, implicating an adaptation of neutrophils to different microvascular environments. These quantitative analyses were largely consistent with our observations (Figure 1F). For example, in the lung, 75% of neutrophils showed a prolonged transition time (Figure 1G and Supplemental Figure 1D, cluster 0). The migration trajectories of these neutrophils were tortuous, and many neutrophils showed dynamic deformation (SD of the cell area, height-to-length [H/L] ratio of the cell, and SD of the oblate ellipticity parameter), which is consistent with the fact that the diameters of lung capillaries are smaller than 5 μm and neutrophils have to deform to squeeze through the capillary (Supplemental Figure 2, A and B). In human lung biopsy tissue, we also observed deformed neutrophils inside capillaries, whereas neutrophils inside larger vasculatures were mostly spherical (Supplemental Figure 2C). On the other hand, the vessel diameters in the liver and spleen were significantly larger than those in the lungs (Supplemental Figure 2D). Approximately 80% of neutrophils in the liver were moving within the vessels with a short transition time and a more straightforward trajectory (cluster 2), whereas a minor subset showed deformation. The spleen harbors an open circulation system (8), which likely explains the observation that a small portion of neutrophils resided within the parenchyma with static behavior and almost spherical cellular morphology (cluster 3). Taken together, we showed that neutrophils could rapidly adapt to different vascular structures and change their behavioral and morphological properties in physiological states. We focused on pulmonary neutrophils, as their abundance and prolonged transition duration suggest that the lung vascular beds may serve as a unique niche for these cells.

### Neutrophils display lung-specific signaling and gene expression patterns at steady state.

**Figure 2:**
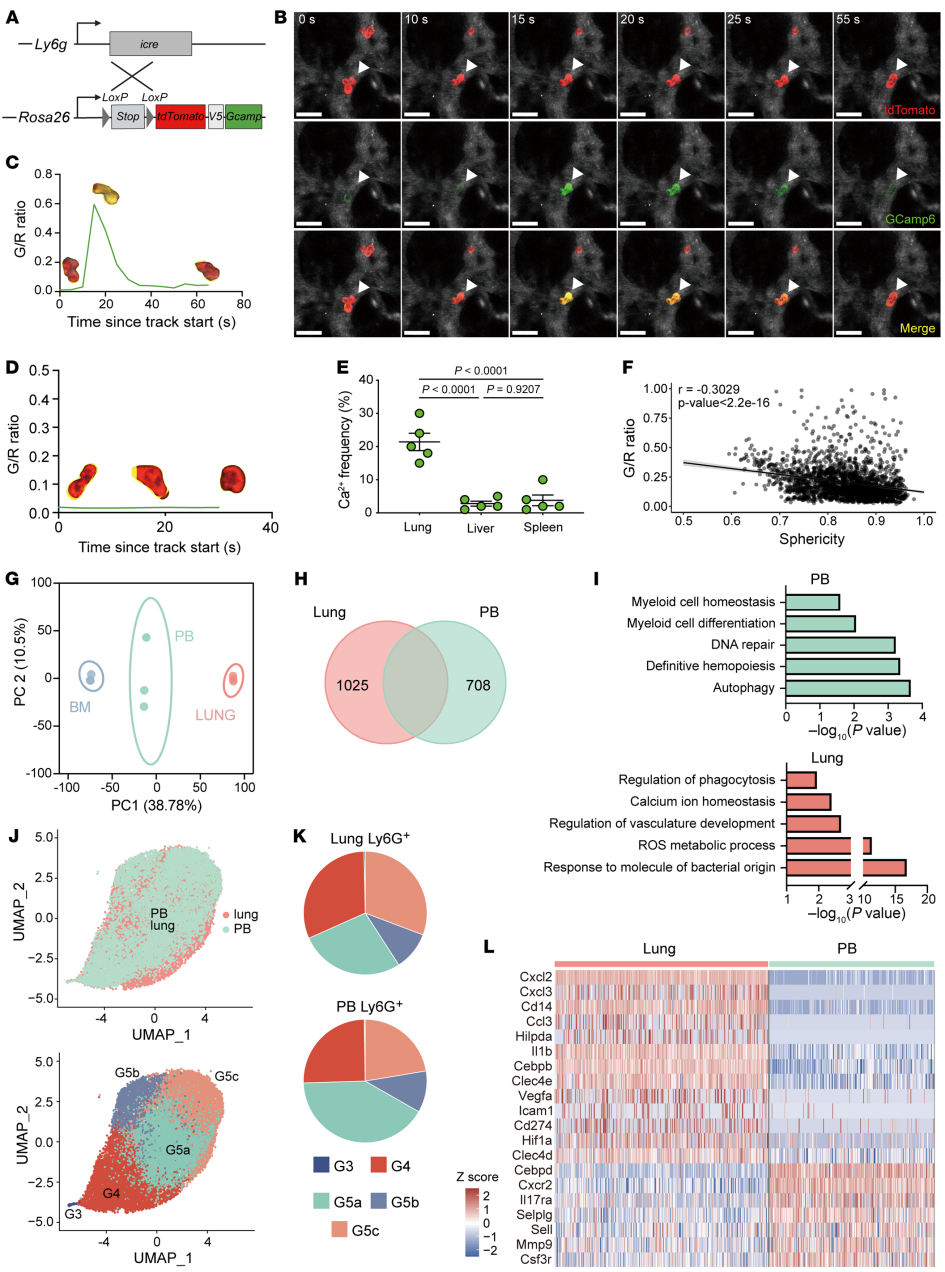
Neutrophils display lung-specific signaling and transcriptome under normal conditions. (**A**) Construction strategy of neutrophil calcium reporter mice. (**B**) Time-lapse images showing a single calcium event in neutrophils in the lung. Scale bars: 30 μm. (**C**) MFI time plot of neutrophil displaying a short Ca^2+^ transient. Images of the Ca^2+^ signals of the cells are transposed onto the plots. (**D**) MFI time plot of neutrophil displaying no Ca^2+^ signal. (**E**) Frequency of Ca^2+^ signals in neutrophils from different tissues. Data were calculated as the percentage of neutrophils displaying Ca^2+^ in total neutrophils during the same recording time. *n* = 5 mice. (**F**) Correlation between the G/R ratio and sphericity of neutrophils in vivo. (**G**) Total transcripts of sorted BM, lung, and PB neutrophils are presented in a PCA plot. *n* = 3 mice. (**H**) Venn diagram representing DEGs between lung and PB neutrophils. (**I**) Pathway analysis of DEGs between lung and PB neutrophils. (**J**) UMAP of 20,941 neutrophils from PB (9,118 neutrophils) and lung (11,823 neutrophils), colored by sample origin (upper panel) or cell type (lower panel). (**K**) Proportions of the 5 neutrophil clusters in lung and PB samples. (**L**) Heatmap showing row-scaled expression of top DEGs from scRNA-Seq data for each averaged tissue profile. Data in **E** indicate the mean ± SEM. Statistical significance was determined by 1-way ANOVA with Tukey’s multiple-comparison test.

Calcium (Ca^2+^) signaling has long been associated with multiple key immunological events, including chemotaxis, phagocytosis, and activation (9). The different migratory behaviors might reflect differences in intracellular signaling dynamics during neutrophil circulation in tissues. To visualize the calcium signaling, we induced expression of the genetically encoded Ca^2+^ indicator Salsa6f (a fusion protein in which tdTomato is linked to the Ca^2+^ indicator GCaMP6f) specifically in neutrophils (*Ly6g^Salsa6f^*) (Figure 2A). We used the GCaMP6f/tdTomato (G/R) ratio to determine the cytosolic Ca^2+^ levels independent of probe concentration or cell movement. Subsequently, we performed simultaneous calcium, kinetics, and morphology imaging of neutrophils in various tissues. Calcium (Ca^2+^) signaling was detected in neutrophils within the lung, characterized by an augmentation in the intensity of green fluorescence (GCaMP6f) overlaid on red fluorescence (tdTomato) (Figure 2B and Supplemental Video 2). Subsequently, we performed the ratiometric analysis of GCaMP6f over tdTomato to subtract out fluctuations in green fluorescence due to cell movement. The representative analysis revealed that neutrophils had frequent, short Ca^2+^ transients (<15 seconds) (Figure 2C), whereas neutrophils over the observed duration exhibited no Ca^2+^ spikes (Figure 2D). Compared with the lung, Ca^2+^ signaling events of neutrophils in liver and spleen were less frequent (Figure 2E and Supplemental Video 3). In the spleen, most stationary neutrophils remained spherical and did not show Ca^2+^ signals over time. Ca^2+^ signaling was prevalent in neutrophils as they circulated through lung capillaries but not in the liver, indicating that the Ca^2+^ signaling observed in the lungs was unlikely to be associated with cell movement (Figure 2E). We performed a correlation analysis of kinetics and morphological characteristics with Ca^2+^ signal intensities. Neutrophils that were less spherical showed stronger Ca^2+^ signals (Figure 2F and Supplemental Figure 3A), whereas there was no direct correlation between Ca^2+^ signal intensity and cell speed or other parameters (Supplemental Figure 3B). This suggests that the Ca^2+^ signal was specifically associated with neutrophil deformation. Spontaneous and evoked Ca^2+^ transients regulate gene expression and functions in neutrophils (10). Therefore, we hypothesize that the calcium influx resulting from neutrophil deformation could initiate downstream signaling cascades, leading to the activation of transcriptional programs.

At steady state, approximately 99% of neutrophils are intravascular (Supplemental Figure 4, A and B). Interestingly, the majority of pulmonary neutrophils remained in the vasculature after a thorough perfusion, whereas RBCs were completely cleared out (Supplemental Figure 4, C and D). We isolated neutrophils from thoroughly perfused lung, PB, and bone marrow (BM) to perform bulk RNA-Seq analysis. Principal component analysis (PCA) of gene expression showed that neutrophils clustered according to tissue and lung neutrophils were at a greater distances from BM neutrophils compared with blood neutrophils (Figure 2G). Since neutrophils are generated in the BM before being released into circulation, our subsequent analysis focused exclusively on neutrophils obtained from the blood and lungs. Hundreds of differentially expressed genes (DEGs) were observed in each tissue neutrophil (Figure 2H and Supplemental Figure 5A). Gene ontology (GO) analysis revealed that the pulmonary neutrophils exhibited positive enrichment in pathways associated with bactericidal function, such as response to bacterial molecules, ROS metabolic processes, and regulation of phagocytosis. Additionally, vasculature development pathways were also enriched in pulmonary neutrophils (Figure 2I). We have selected several genes with potential functional relevance for further validation. Comparing neutrophils isolated from PB and lung in the same mouse revealed that these tissue-specific signatures were consistently different across individual mice, as measured by quantitative PCR (qPCR) (Supplemental Figure 5B). Similarly, neutrophil isolated from human lung tissue showed higher expression of genes such as *VEGFA*, *IL1B*, *CXCL1*, *CXCL2*, *CCL3*, and *IL6* compared with those from matched PB (Supplemental Figure 5C). At the protein level, we also detected a higher level of VEGFA, IL-6, IL-1B, and CD274 in mouse lung neutrophils than in BM or PB neutrophils (Supplemental Figure 5D). The transcriptional heterogeneity of neutrophils among different tissues prompted us to further explore neutrophil diversity in PB and lung using single-cell transcriptome profiling. We used previously reported signature genes to distinguish each subpopulation and found that polymorphonuclear neutrophils (PMNs) in circulating blood and lung could be classified into 5 cell subgroups, including the less mature G3 and G4 subsets and the mature G5a–c subsets (11). This finding highlights the conserved nature of these neutrophil subpopulations (Figure 2J). However, the proportions of G4 and G5c were higher in the lung compared with blood, whereas the G5a proportion was higher in the blood (Figure 2K). Although the neutrophil subpopulations were conserved, many genes underwent profound changes in the lung (Figure 2L). Nonetheless, most of these lung-specific signatures were evenly found in each G5 subpopulation and slightly lower in the G4 subset (Supplemental Figure 6). The G4 subset was previously described as newly mature neutrophils in the BM, indicating that the G4 subset may be less mature than the peripheral G5 subsets. However, it is unlikely that lung-specific gene signatures are restricted to any subpopulation. Consequently, in the subsequent experiments, we studied lung neutrophils as a whole population. Collectively, we demonstrated that calcium signaling was linked to neutrophil deformation as these cells circulated through lung capillaries, which potentially contributed to tissue-specific transcriptional signatures in lung neutrophils.

### PIEZO1 regulates pulmonary neutrophil behaviors and transcriptional profile.

**Figure 3:**
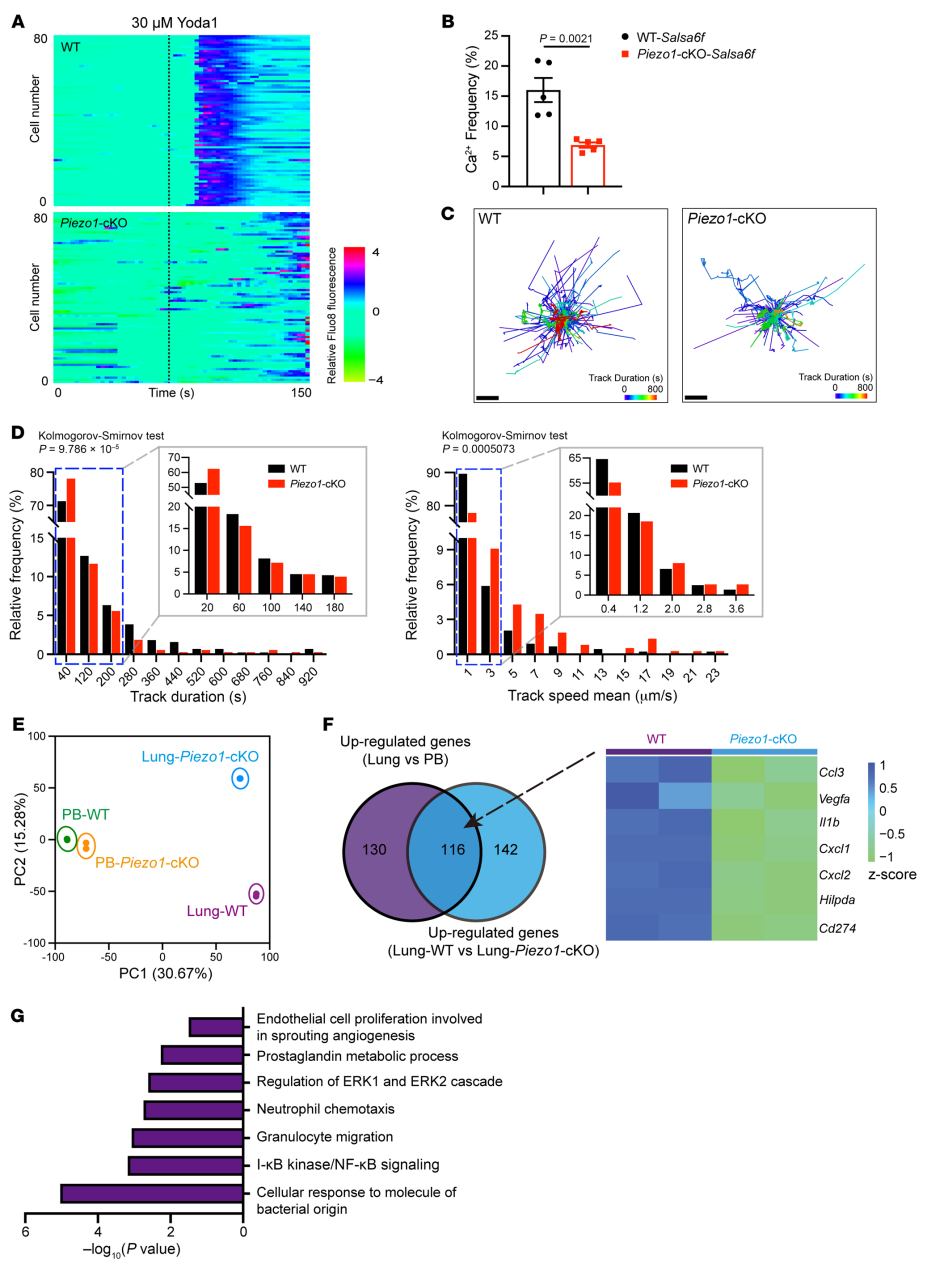
PIEZO1 regulates pulmonary-specific neutrophil behavior and the transcriptome. (**A**) Heatmap of normalized calcium fluorescence versus time for each BM neutrophil isolated from WT and *Piezo1*-cKO mice. (**B**) Frequency of Ca^2+^ signals in neutrophils, as assessed by intravital imaging of lungs from WT *Salsa6f* and *Piezo1*-cKO-*Salsa6f* mice. *n* = 5. (**C**) Migration trajectory of WT and *Piezo1*-cKO neutrophils in the lungs. Bars indicate track duration. (**D**) Distribution frequency of track duration and average track velocity of WT and *Piezo1*-cKO neutrophils in the lung. (**E**) PCA of gene expression data of neutrophils isolated from the blood and the lungs of WT and *Piezo1*-cKO mice. (**F**) Venn diagram showing the commonly upregulated genes across the specified comparisons. Genes with potential functional significance were selected, and their expression levels are presented in a heatmap. The value is *z* score calculated from scaling log_2_ (transcripts per million [TPM]) values. (**G**) Pathway analysis of DEGs between WT and *Piezo1*-cKO neutrophils in lung. Data in **B** indicate the mean ± SEM. Statistical significance was determined by unpaired, 2-tailed Student’s *t* test (**B**) and 2-sample Kolmogorov-Smirnov test (**D**).

Our imaging data revealed that neutrophils undergo frequent deformations as they move through the pulmonary circulation, repeatedly experiencing physical strain in numerous small vessels. The observed phenomenon prompted us to explore whether mechanical sensing by neutrophils might play a role in shaping lung-specialized neutrophil characteristics. Consequently, we examined the expression levels of known mechanosensitive ion channels in neutrophils. *Piezo1*, and to a lesser extent, *Trpv4* were abundantly expressed in neutrophils (Supplemental Figure 7A). The expression of PIEZO1 in lung neutrophils was confirmed using *Piezo1-tdTomato* mice, in which a PIEZO1-tdTomato fusion protein is expressed (Supplemental Figure 7B). In an attempt to generate conditional *Piezo1*-deficient animals, mice expressing Cre recombinase directed by the *Vav1*, *Lyz2*, or *S100a8* promoters were crossed with mice expressing homozygous loxP sites around exons 20–23 of *Piezo1*. However, only with *Vav1-Cre*, *Piezo1* was efficiently deleted from neutrophils at the transcription and protein levels (Supplemental Figure 7, C and D). This may be due to the differential kinetics of gene expression during neutrophil development. Analysis of available datasets revealed that both *Vav1* and *Piezo1* reached their peak expression levels in early progenitors, whereas *Ly6g*, *S100a8*, and *Lyz2* were all expressed at late stages (Supplemental Figure 7E). Live-cell imaging revealed that PIEZO1 agonist Yoda1 stimulation triggered a transient calcium influx in WT neutrophils but not *Piezo1*^DVav1^ conditional-KO (*Piezo1*-cKO) neutrophils (Figure 3A). The whole-cell patch-clamp recording revealed the neutrophil response to pressure, which was completely abolished in the absence of Piezo1 (Supplemental Figure 7, F and G). To determine whether PIEZO1-mediated mechanical sensing contributes to neutrophil dynamics in vivo, we performed intravital imaging to compare migratory behaviors, morphology, and Ca^2+^ signaling of pulmonary neutrophils in WT and *Piezo1*-cKO mice that also express Gcamp (Supplemental Video 4). Both WT and *Piezo1*-cKO neutrophils had similar morphology features, indicating that the deformability of neutrophil was not affected by PIEZO1 (Supplemental Figure 8A). *Piezo1*-deficient neutrophils displayed a reduced frequency of Ca^2+^ signaling during their transit through the lung (Figure 3B). Analysis of the migration trajectory indicated that WT neutrophils persisted in the lung for a longer duration when compared with the *Piezo1*-cKO neutrophils (Figure 3C). The distribution of neutrophil transit durations revealed a higher frequency of brief transit times in *Piezo1*-cKO mice. Correspondingly, a larger proportion of neutrophils in WT mice traversed at slower speeds compared with those in *Piezo1*-cKO mice (Figure 3D). We further performed an adoptive transfer experiment to rule out the possibility that other cell types may influence neutrophil behavior. The migration patterns of the transferred WT and *Piezo1*-cKO neutrophils were similar to those seen in the original WT and *Piezo1*-cKO mice (Supplemental Figure 8, B–D). The changes in these dynamic features and intracellular signaling events highlight the essential role of mechanical sensing via PIEZO1 in regulating neutrophil behaviors in the lung. However, WT and *Piezo1*-cKO neutrophils exhibited similar dynamics and morphological characteristics in the liver and spleen (Supplemental Figure 8, E and F). To further explore whether PIEZO1-mediated mechanical sensing influences lung-specific transcriptional signatures in neutrophils, we performed bulk RNA-Seq in lung and blood neutrophils isolated from WT and *Piezo1*-cKO mice. The PCA analysis revealed a profound transcriptional difference between WT and *Piezo1*-cKO neutrophils in the lung compared with those in the blood (Figure 3E). Notably, there were 116 shared upregulated genes between upregulated genes in lung versus PB neutrophils and those in WT versus *Piezo1*-cKO pulmonary neutrophils. Several lung-specific neutrophil genes with potential functional relevance were highly expressed in WT neutrophils compared with those in *Piezo1*-cKO neutrophils (Figure 3F). GO analysis revealed that PIEZO1 influenced several biological pathways, many of which were also enriched in lung neutrophils compared with blood neutrophils (Figure 3G). The most enriched pathway was related to EC proliferation and angiogenesis, suggesting that PIEZO1 plays a key role in the proangiogenic specialization of lung neutrophils.

### Rapid neutrophil reprogramming driven by PIEZO1 activation.

**Figure 4:**
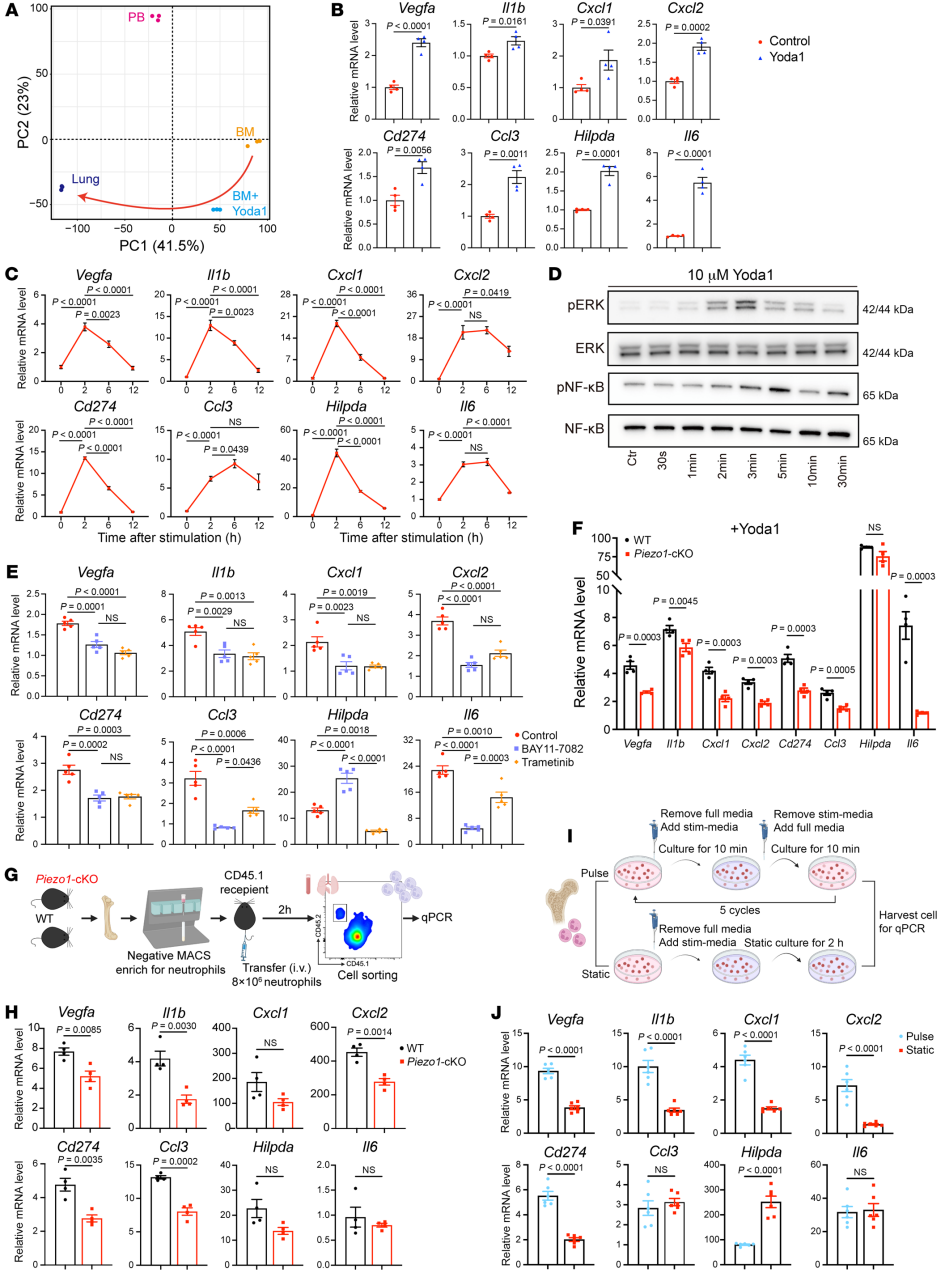
PIEZO1 activation reprograms neutrophils to acquire the lung signature. (**A**) Total transcripts of sorted BM, BM stimulated with Yoda1 for 2 hours (BM+Yoda1), lung, and PB neutrophils are presented in a PCA plot. *n* = 3. (**B**) BM-derived neutrophils were stimulated with Yoda1 for 2 hours. Relative mRNA expression of the indicated genes in neutrophils was determined by qPCR. *n* = 4. (**C**) BM-derived neutrophils were stimulated with Yoda1 for the indicated durations. Relative mRNA expression of the indicated genes in neutrophils was determined. *n* = 3–6. (**D**) Immunoblot analysis of phosphorylated ERK (pERK), pNF-κB, total ERK, and total NF-κB in BM-derived neutrophils treated with Yoda1 for indicated durations. Blots are representative of 3 independent experiments. (**E**) BM-derived neutrophils were pretreated with either trametinib (ERK inhibitor) or BAY 11-7082 (NF-κB inhibitor) for 30 minutes before stimulation with Yoda1 for 2 hours. Relative mRNA expression of the indicated genes in neutrophils was determined. *n* = 5. (**F**) BM-derived neutrophils from WT and *Piezo1*-cKO mice were treated with Yoda1 for 2 hours. Relative mRNA expression of the indicated genes in neutrophils was determined *n* = 4. (**G**) Schematic of experimental workflow. MACS, magnetic-activated cell sorting. (**H**) BM-derived neutrophils from the indicated mice were i.v. injected into CD45.1 recipient mice. Two hours later, the transferred neutrophils were sorted from the indicated tissue and subjected to qPCR analysis. Relative mRNA expression of the indicated genes in neutrophils was determined. *n* = 4. (**I**) Schematic of experimental workflow. stim-media, stimulated media.(**J**) BM-derived neutrophils were pulse treated as indicated in **I**. Relative mRNA expression of the indicated genes in neutrophils was determined. *n* = 6. Data in **B**, **C**, **E**, **F**, **H**, and **J** indicate the mean ± SEM. Statistical significance was determined by 1-way ANOVA with Tukey’s multiple-comparison test (**C** and **E**), multiple Student’s *t* test (**F**), and unpaired, 2-tailed Student’s *t* test (**B**, **H**, and **J**).

The above results suggest that neutrophils were able to reprogram when circulating into different tissues and that neutrophil reprogramming in lung is likely to be driven by PIEZO1 signaling. To get a broader understanding of how PIEZO1 activation drives lung-specific neutrophil signatures at the transcriptional level, we stimulated neutrophils purified from BM with Yoda1. Bulk RNA-Seq analysis demonstrated the global transcriptional changes of BM-derived neutrophils upon stimulation with Yoda1 toward pulmonary neutrophils (Figure 4A). We chose the expression of a subset of the lung-associated genes as an indicator of neutrophil reprogramming. We observed the expression of these genes to be profoundly upregulated upon Yoda1 stimulation, as measured by qPCR (Figure 4B). Similarly, the expression of lung-associated signatures was also increased in neutrophils from human PB upon stimulation with Yoda1 (Supplemental Figure 9). The upregulation of most genes peaked at 2 hours and gradually decreased or remained unchanged over time (Figure 4C). Yoda1 stimulation induced rapid phosphorylation of ERK, which has been reported to play a critical role in mechanotransduction in different cell types (Figure 4D). NF-κB is an important transcription factor that can be activated by ERK. Indeed, phosphorylation of NF-κB was also induced upon Yoda1 stimulation (Figure 4D). Pharmacological inhibition of ERK or NF-κB resulted in reduced expression of lung-associated genes in neutrophils (Figure 4E). The upregulation of lung-associated neutrophil genes in response to Yoda1 stimulation was specific to PIEZO1. In the absence of *Piezo1*, the expression of these genes was significantly reduced upon Yoda1 stimulation (Figure 4F). To further validate the PIEZO1-dependent acquisition of lung-specific signatures in vivo, BM neutrophils from WT or *Piezo1*-cKO mice were transferred into naive CD45.1 recipient mice (Figure 4G). The exogenous neutrophils in the lungs rapidly acquired lung-associated signatures as early as 2 hours after transfer, which were significantly decreased in the absence of PIEZO1 (Figure 4H). As revealed by live-cell imaging, PIEZO1 activation elicited transient Ca^2+^ influx upon Yoda1 stimulation, whereas lung neutrophils displayed multiple Ca^2+^ transients in vivo, suggesting that PIEZO1 was repeatedly activated. Therefore, we developed a pulsed stimulation system by repetitively adding Yoda1 to neutrophil culture (Figure 4I). The pulse stimulation resulted in significantly higher expression of lung-associated genes such as *Vegfa*, *Il1b*, *Cxcl1*, and *Cxcl2* (Figure 4J). Together, these data demonstrated that activation of PIEZO1 directly reprogrammed neutrophils to express proangiogenic genes.

### Confinement recapitulates the pulmonary signature in neutrophils.

**Figure 5:**
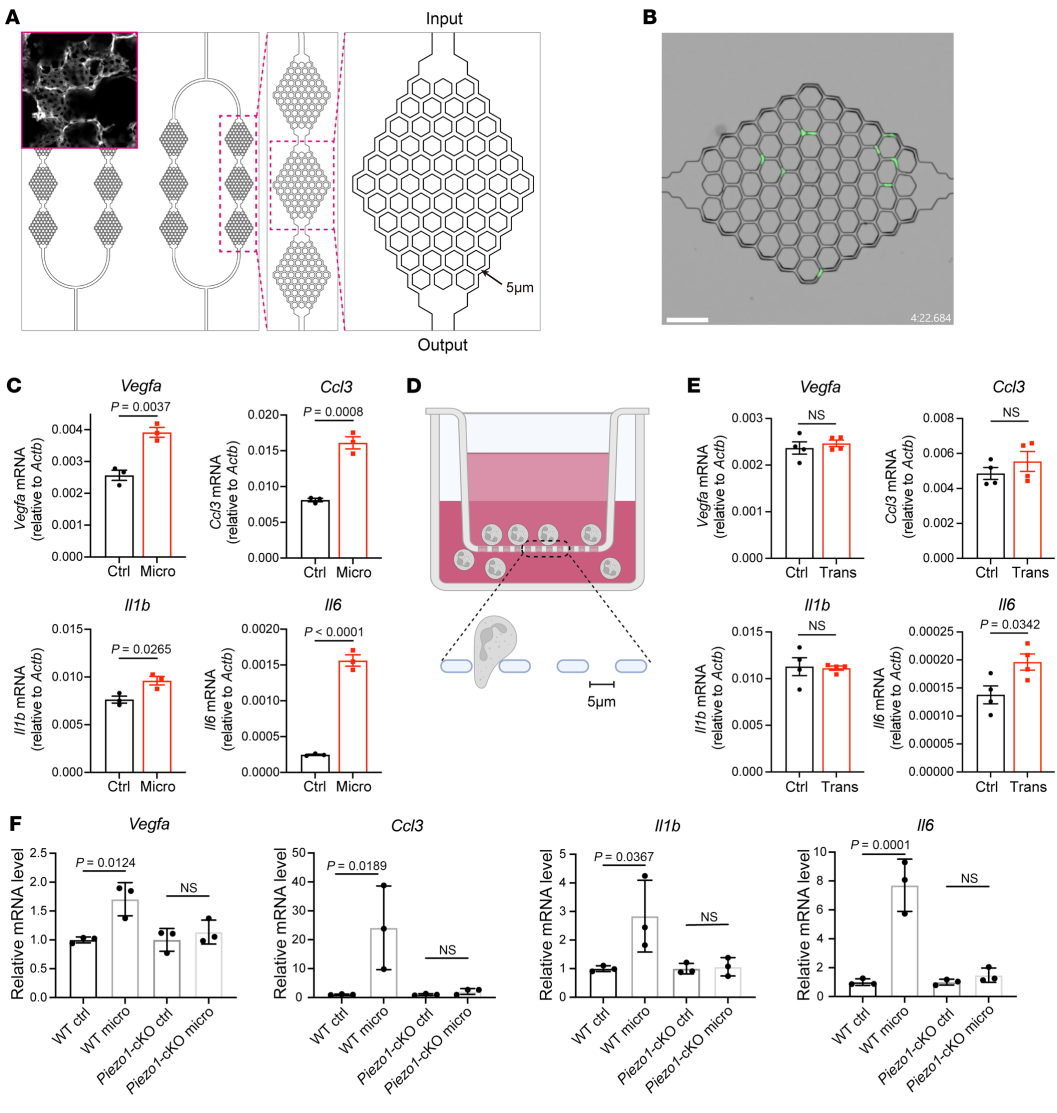
Repetitive neutrophil constriction is sufficient to instruct lung-associated signatures via PIEZO1. (**A**) Design of the branching microfluid mimetic of the pulmonary vasculature. Inset in the left panel is the confocal image of lung capillary network stained with CD31. Original magnification, ×20. (**B**) Image of CM-FDA–labeled neutrophils (green) perfused through a microfluid device. Scale bar: 50 μm. (**C**) Expression of the indicated genes in neutrophils that passed through microfluid (Micro) and in neutrophils kept in the same buffer for the same duration (Ctrl). *n* = 3. (**D**) Illustration showing spontaneous migration of neutrophils through a Transwell with a pore diameter of 5 μm. (**E**) Expression of the indicated genes in neutrophils that passed through the Transwell (Trans) and in neutrophils remaining in the upper chamber (Ctrl). *n* = 4. (**F**) Expression of the indicated genes in WT and *Piezo1*-cKO neutrophils that passed through microfluid (micro) and in neutrophils kept in the same buffer for the same duration (Ctrl). *n* = 3. Data in **B**, **D**, and **E** indicate the mean ± SEM. Statistical significance was determined by unpaired, 2-tailed Student’s *t* test.

To mimic the repeated confinement that neutrophils encounter in the lung, we used a microfluidic microvasculature mimetic to test whether neutrophil trafficking through known pore diameters could induce changes in gene expression (Figure 5A). In particular, we examined the proangiogenic genes that were upregulated in pulmonary neutrophils (*Vegfa*, *Ccl3*, *Il1b*, and *Il6*). The minimum gaps (5 μm) were smaller than the diameter of cells but matched the size of the pulmonary capillaries. Neutrophils that were perfused to migrate through microfluidic channels exhibited significant deformation and were notably retained within the channels (Supplemental Video 5 and Figure 5B). We found that neutrophils passing through the microfluidic device showed higher expression of *Vegfa*, as well as other genes such as *Ccl3*, *Il1b*, and *Il6* compared with neutrophils that did not pass through the microfluid device (Figure 5C). Interestingly, when neutrophils were allowed to spontaneously migrate through a single 5 μm porous membrane using the Transwell system, only *Il6* showed a moderate upregulation, whereas the expression of *Vegfa*, *Ccl3*, and *Il1b* remained unchanged (Figure 5, D and E). The above data suggested that neutrophils only upregulated these genes when experiencing repetitive constriction, similar to what happens in the lung when neutrophils migrate through the capillaries. To determine whether PIEZO1 activation contributes to the upregulation of the selected genes, we perfused neutrophils from WT or *Piezo1*-cKO mice through a microfluidic microvasculature mimetic. The data suggested that the upregulation of these genes was only seen in WT but not *Piezo1*-cKO neutrophils after multiple constriction (Figure 5F). Taken together, our data indicate that repeated activation of PIEZO1 when passing through capillary constrictions resulted in the expression of proangiogenic signatures in neutrophils.

### PIEZO1-mediated mechanical sensing enhances bactericidal capacity.

**Figure 6:**
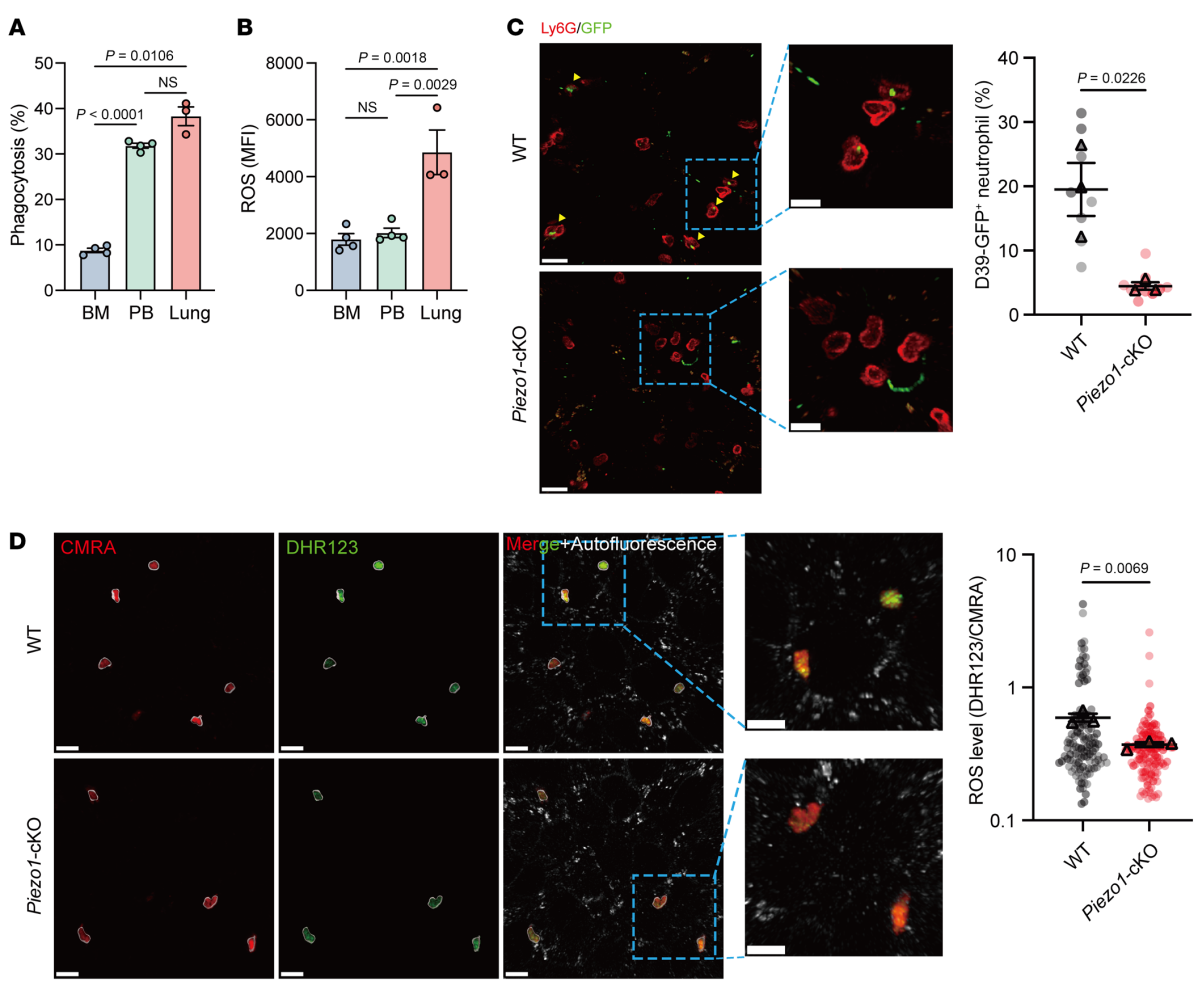
PIEZO1-mediated mechanical sensing prepares neutrophils for host defense. (**A** and **B**) Phagocytosis of pHrodo-labeled *E. coli* bioparticles (**A**) and ROS production (**B**) by sorted neutrophils from the indicated tissues. *n* = 3–4 mice. (**C**) Representative images from intravital imaging and quantification of bacteria phagocytosis by neutrophils in vivo. Scale bars: 20 μm. *n* = 3 mice. (**D**) Representative images from intravital imaging and quantification of ROS generation by neutrophils in vivo. Scale bars: 20 μm. *n* = 4 mice. The *y*-axis is plotted on a logarithmic scale. Data are indicate the mean ± SEM. Statistical significance was determined by 1-way ANOVA with Tukey’s multiple-comparison test (**A** and **B**) and unpaired, 2-tailed Student’s *t* test (**C** and **D**).

PIEZO1 activation has been shown to enhance phagocytosis and ROS production in macrophages through a Ca^2+^-dependent manner (12, 13). Indeed, in vitro stimulation of neutrophils isolated from lung, PB, and BM revealed higher phagocytic capability and more ROS generation in lung neutrophils (Figure 6, A and B). The above results indicated that, while in the vasculature, the neutrophils were already predisposed for a more effective antibacterial response in the lungs. We therefore investigated the role of PIEZO1 in the modulation of the above functions. We use intravital imaging to evaluate phagocytosis and ROS generation in WT and *Piezo1*-cKO mice in vivo. *Streptococcus pneumoniae* (D39-GFP) was i.v. injected to visualize their capture by pulmonary neutrophils. We observed more neutrophils capturing bacteria in WT mice than in *Piezo1*-cKO mice (Figure 6C). To visualize ROS generation in vivo, ROS probe DHR123-labeled neutrophils were transferred to D39-infected mice. Intravital imaging revealed a stronger DHR123 signal in WT neutrophils than in *Piezo1*-cKO neutrophils (Figure 6D). The aforementioned findings indicate that pulmonary neutrophils had a predisposition for host defense function, a process mediated by Piezo1 signaling.

### Intravascular activation of PIEZO1 induces VEGFA in neutrophils to sustain vascular homeostasis.

**Figure 7:**
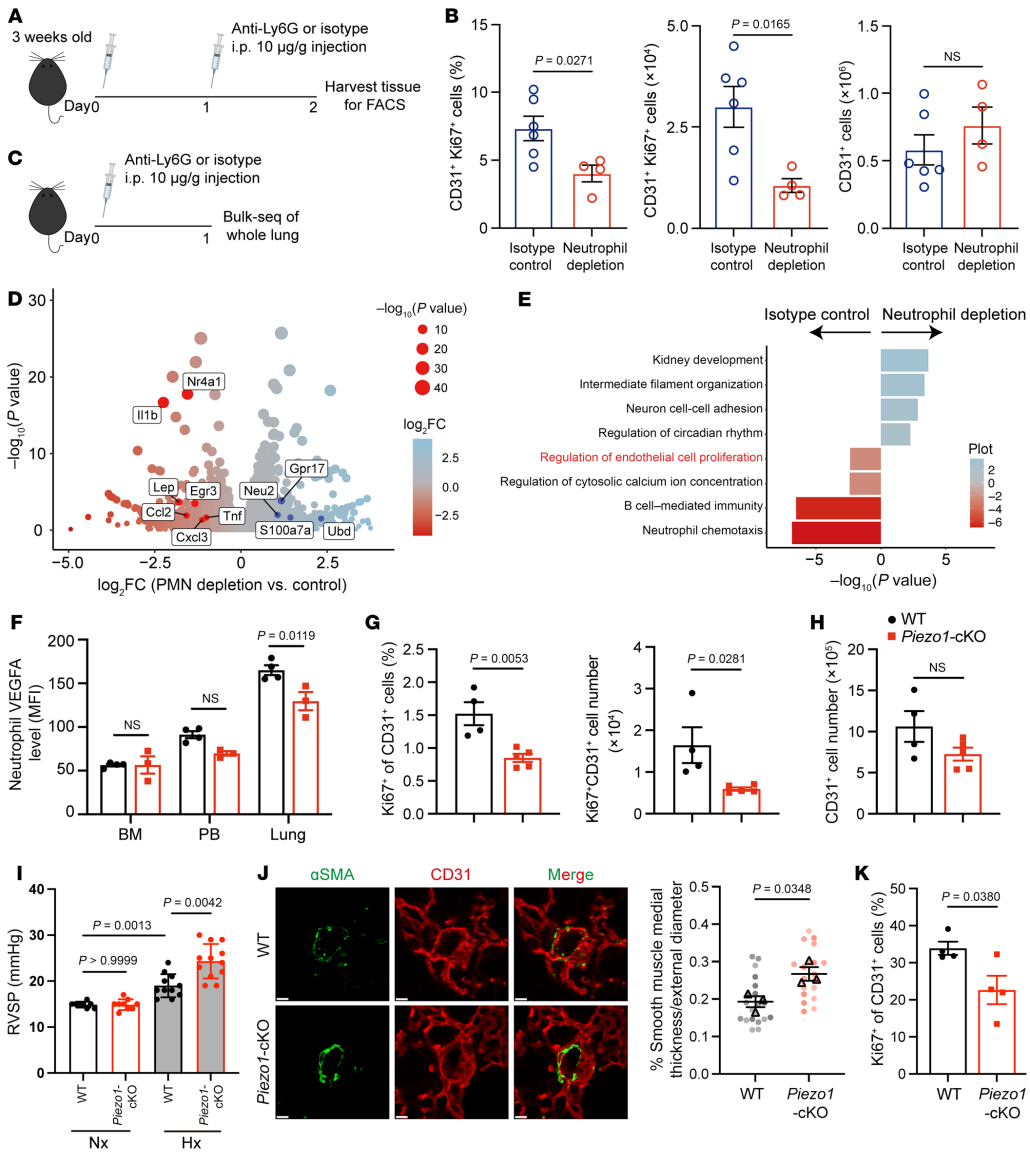
Intravascular activation of PIEZO1 induces VEGFA in neutrophils to sustain vascular homeostasis. (**A**) Schematic of the experimental workflow. (**B**) Flow cytometric analysis of the percentage and number of CD31^+^Ki67^+^ cells and the total number of CD31^+^ cells in control and neutrophil-depleted lungs. *n* = 4 (neutrophil depletion); *n* = 6 (isotype control). (**C**) Schematic of the experimental workflow. (**D**) Volcano plot showing DEGs between control and neutrophil-depleted lungs. Transcripts significantly upregulated in either PMN-depleted or control lungs are colored in blue and red, respectively (log_2_ fold change ± 0.5 and adjusted *P* < 0.05). (**E**) GO enrichment analysis was performed on the DEGs. Blue indicates enrichment in lungs from neutrophil-depleted mice, while red indicates enrichment in lungs from control mice. (**F**) Intracellular VEGFA levels in neutrophils from the indicated tissues. *n* = 3–4 mice. (**G**) Percentage and absolute count of proliferating ECs in lungs from 3-week-old mice. *n* = 4 WT mice; *n* = 5 *Piezo1*-cKO mice. (**H**) Absolute count of total ECs in lung from 3-week-old mice. *n* = 4 WT mice; *n* = 5 *Piezo1*-cKO mice. (**I**) RVSP in normoxia (Nx) and hypoxia (Hx) for 3 weeks. *n* = 9 (Nx), *n* = 11 (Hx, WT); *n* = 12 (Hx, *Piezo1*-cKO). (**J**) Representative images and quantification of α-SMA–stained arterioles. Scale bars: 10 μm. *n* = 3. (**K**) Flow cytometric analysis of proliferating ECs in lung after 3 weeks of hypoxia. *n* = 4. Data in **B** and **F**–**K** are shown as the mean ± SEM. Statistical significance was determined by 1-way ANOVA with Tukey’s multiple-comparison test (**I**) and unpaired, 2-tailed Student’s *t* test (**B**, **F**, **G**, **H**, **J**, and **K**).

An important function previously documented to be specialized for lung neutrophils is their contribution to pulmonary vascularization (4). Similar to the previous report, neutrophil depletion at an early age resulted in a significant loss of the proliferative capacity of ECs (Figure 7, A and B). To investigate whether neutrophil depletion may have a broader influence in the lung tissue, we performed bulk RNA-Seq of the entire lung tissue in neutrophil-depleted mice (Figure 7, C and D). Antibody treatment efficiently depleted neutrophils without affecting monocytes in the PB and lung (Supplemental Figure 10, A and B). The analysis of transcriptomics data revealed that neutrophil depletion affected many genes that could be classified into specific pathways, including regulation of ECs (Figure 7E). To explore the role of PIEZO1 in mediating angiogenic function, we focused on VEGFA, which was highly expressed in lung neutrophils but was downregulated in the absence of *Piezo1*. At the protein level, VEGFA levels in lung and PB neutrophils from *Piezo1*-cKO mice were notably lower than those in WT neutrophils (Figure 7F). Consequently, compared with young WT mice, *Piezo1*-cKO mice showed impaired proliferation in lung ECs (Figure 7, G and H). In addition, mice with *Vegfa* deleted in neutrophils phenocopied the proliferation defect of lung ECs as observed in *Piezo1*-cKO mice (Supplemental Figure 10, C–F), suggesting that VEGFA was the primary target of PIEZO1-mediated proangiogenic function. To rule out the possibility that PIEZO1 in non-neutrophil cells might also influence pulmonary physiology, we conducted an adoptive transfer experiment. Using *Ly6g-DTR* mice, we administered diphtheria toxin to mice at an early age to achieve complete neutrophil depletion. Neutrophils from WT and *Piezo1*-cKO mice were transferred into these neutrophil-depleted animals for 48 hours, and pulmonary EC proliferation was assessed. Neutrophil depletion led to reduced EC proliferation, which was rescued by the transfer of WT neutrophils but not by *Piezo1*-cKO neutrophils (Supplemental Figure 10G). To test whether PIEZO1-mediated VEGFA production in neutrophils also plays a role in diseases characterized by disordered vascular homeostasis, we examine EC proliferation in WT and *Piezo1*-cKO mice exposed to LPS-induced acute lung injury or chronic hypoxia–induced pulmonary hypertension. Previous studies have characterized an important role of VEGFA in mediating vascular remodeling in both models (14, 15). Ten days after LPS-induced acute lung injury, *Piezo1*-cKO mice showed impaired proliferation in lung ECs (Supplemental Figure 10H). After 3 weeks’ exposure to hypoxia, both WT and *Piezo1*-cKO mice exhibited elevated right ventricular systolic pressure (RVSP). Compared with WT mice, we found that RVSP was significantly greater in *Piezo1*-cKO mice (Figure 7I). Immunofluorescence staining for α–smooth muscle actin (α-SMA) in the medial area of the arterioles revealed increased α-SMA staining in the *Piezo1*-cKO compared with WT mice (Figure 7J). Hypoxia exposure resulted in proliferation of ECs, and there were fewer proliferating ECs in *Piezo1*-cKO mice than in WT mice (Figure 7K). Taken together, a decreased angiogenic response due to *Piezo1* deficiency in neutrophils impaired EC proliferation in both acute and chronic pulmonary vascular dysfunction. Overall, these findings indicate that PIEZO1-mediated proangiogenic function in neutrophils was essential for pulmonary vascular homeostasis.

## Discussion

Neutrophil heterogeneity and plasticity have recently gained attention (16). Emerging technology, including single-cell sequencing and CyToF, has been used to demonstrate different subsets of neutrophils in mice and humans based on differences in transcript and protein levels (4, 11, 17). Although a vast degree of heterogeneity between neutrophils from different tissues has been recognized, how tissues instruct their specific reprogramming is poorly defined. Neutrophils are characterized by a high degree of dynamism and are exposed to diverse tissue environments while circulating. The majority of neutrophils remain in a state of equilibrium within the bloodstream, with the exception of those located in the spleen, lymph nodes, and intestinal tissue (18). It is unlikely that neutrophils will receive as many “imprinting” signals as other resident cells, such as macrophages from the local parenchyma, because the blood vessel barrier physically separates them.

On the other hand, vascular architectures and hemodynamics might be critical in determining neutrophil identity. Here, we performed high-throughput intravital imaging studies to compare neutrophil migration behavior in different organs. To this end, we identified different neutrophil behaviors in the lung on the basis of their kinetics and morphology. This “behavior landscape” approach, originally introduced by Crainiciuc et al. (7), enabled an unbiased analysis of movement and shape from thousands of individual cells. While the previous study concentrated on inflammation, our study focused on comparing cellular behaviors across different tissues in a steady-state condition. The unique behavioral features of neutrophils in the lung further encouraged us to characterize the specific transcriptional features of neutrophils in the lungs using bulk RNA-Seq and single-cell RNA-Seq (scRNA-Seq). These complementary assays provided consistent results and further confirmed the specific signatures of neutrophils in the lung. Thus, there is an intense interest in integrating data from different layers to gain better insight into the heterogeneity and plasticity of neutrophils in the future.

Large numbers of neutrophils in healthy lung tissue suggest that they perform physiological functions and can adapt to the local environment. Our data showed that neutrophils profoundly changed their transcriptome profiles from the BM to the blood and lungs, indicating that neutrophils underwent tissue adaptation. A previous study suggested that the CXCR4/CXCL12 axis may form instructing regions or niches, where neutrophils receive tissue-derived signals (4). Here, we focused on the unique anatomic structure of lung. The alveoli are the basic working unit for respiratory function in the lungs. A single alveolus is surrounded by approximately 1,000 interconnected short, tubular capillary segments arranged in spheroidal mesh (19). This unique structure guarantees efficient gas exchange. A neutrophil is estimated to pass through approximately 40–100 capillary segments during a single transit from an arteriole to a venule (20). Because of the size mismatch, in at least half of these segments, neutrophils are required to deform to get through. The phenomenon of neutrophil deformation during transit through the pulmonary vasculature has been observed and documented over several decades. However, to our knowledge, a comprehensive investigation into the biological implications of these observed behaviors has yet to be conducted (21, 22). Our study demonstrates the critical role of these changing mechanical properties in shaping neutrophil identities. Several families of mechanically sensitive receptors have been reported to transduce mechanical stimuli into ion currents, thereby regulating physiological processes (23). PIEZO1 has been identified as a component of mechanically activated cation channels in both vertebrates and nonvertebrates (24). *Piezo1* is expressed in a wide range of cell types, where it responds to various biomechanical stimuli and initiates diverse functional outcomes. Previous studies have demonstrated that PIEZO1 in neutrophils can be activated by shear stress– and deformation-induced membrane tension, leading to calcium influx that drives calcium-dependent biological processes. However, the outcomes of these processes differ: shear stress triggers NETosis, whereas deformation promotes NOX4 expression and increases ROS generation (25, 26). In our study, we demonstrated that PIEZO1 activation occurred intravascularly due to the size mismatch between neutrophils and pulmonary capillaries and that this activation enhanced the expression of proangiogenic signatures at steady state. Our study differs from the work by Mukhopadhyay et al. (26), which focused on neutrophil deformation during transmigration under inflammatory conditions. Therefore, these varying outcomes of calcium-dependent processes may result from differences in the duration and amplitude of the calcium signal, as well as the potential influence of other external stimuli acting synergistically on neutrophils. The development of tools and mathematical models capable of estimating in vivo forces will advance our understanding of how various types of forces influence calcium signaling, thereby regulating different biological processes. Our data also suggest that PIEZO1 activation induces pulmonary-specific neutrophils via the ERK and NF-κB pathways, indicating the presence of tonic ERK and NF-κB signaling in neutrophils in the lung. Further study of the role of these tonic signals in regulating neutrophil functions under steady-state conditions will provide a more comprehensive understanding of the contribution of neutrophils to tissue homeostasis and disease.

The function of PIEZO1 in innate immune cells has recently been documented (27). Activation of PIEZO1 in monocytes has been related to increased proinflammatory gene expression in lung infection, sepsis, pulmonary fibrosis, and renal fibrosis (13, 28). PIEZO1-mediated silencing of the retinoblastoma gene *Rb1* drives immunosuppressive myelopoiesis in cancer and infectious disease (10). PIEZO1 can augment CD8^+^ T cell exhaustion via activation of the transcription factor OSR2 in the tumor microenvironment (29). Here, we demonstrated that the lack of *Piezo1* in hematopoietic cells resulted in impaired capillary angiogenesis in vivo and suggest that *Piezo1* on neutrophils was essential in regulating proangiogenic reprogramming to sustain vascular homeostasis. It is important to note that angiogenesis involves multiple processes, with EC proliferation being one critical component. To gain a more comprehensive understanding of the role of PIEZO1-mediated neutrophil reprogramming in this process, further analysis of additional mechanisms, such as tube formation, is essential. In addition, PIEZO1 on other immune cells may also contribute to maintaining vascular homeostasis in the lungs. It is worth noting that the commonly used neutrophil-specific Cre drivers were not effective in depleting *Piezo1* in our experiments, probably because of differences in the expression stages of the genes driving Cre expression and the target genes. Therefore, careful consideration is needed when targeting genes specifically in neutrophils.

Collectively, we show that neutrophils display unique behavioral, transcriptional, and functional signatures in the lungs. These specialized signatures of neutrophils are instructed by local mechanical signals generated when neutrophils migrate through small capillaries, a concept with major possible implications in understanding the local adaptations of the immune adaptations across organs and pathological scenarios.

## Methods

### Sex as a biological variable

All animal studies included equal representations of male and female mice. Both male and female patients were involved for sample collection.

### Animals

*Ly6g-Cre-2A-tdTomato* and *Ly6g-DTR* were purchased from Shanghai Model Organisms. *Piezo1-tdTomato* (strain no. 029214), *Salsa6f* (strain no. 031968), *S100A8-Cre* (strain no. 021614), and *Lyz2-Cre* (strain no. 004781) were purchased from The Jackson Laboratory. *Vegfa^fl/fl^* was purchased from Cyagen Biosciences. The *Piezo1^fl/fl^* (0292143) and *Vav1-iCre* (008610) were from The Jackson Laboratory and were provided by Jianwei Wang from Tsinghua University (Beijing, China) and Zhaoyuan Liu from Shanghai Jiao Tong University School of Medicine (Shanghai, China), respectively. For *Vav1-iCre*, Cre^+^ female mice were mated with Cre^–^ males to avoid nonhematopoietic expression of Cre. The C57BL/6J mice involved in this study were all purchased from SLAC ANIMAL. The mice used in the study were 7- to 12-week-old females or males (no sex difference was observed). The mice were housed under specific pathogen–free conditions at approximately 22°C with humidity set at 40%–70% and a 12-hour light/12-hour dark cycle. It is generally recognized that neutrophils have intrinsic circadian oscillations and that they manifest different phenotypes at varying times. As a result, animal experiments typically started in the morning (around 10 am) except for intravital imaging.

### Human specimens

Paired, noncancerous adjacent lung tissues and PB samples were obtained from 6 patients with lung adenocarcinoma (LUAD) at a very early stage (Supplemental Table 2). Noncancerous adjacent lung tissues (>3 cm from the tumor’s edge) were surgically resected and transferred to RPMI 1640 medium supplemented with 2% FBS and stored at 4°C less than 30 minutes before performing experiments. PB was collected prior to surgery by venipuncture and preserved in EDTA-coated tubes. For human PB–derived neutrophil stimulation, PB samples were obtained from healthy volunteers.

### Intravital imaging and data processing

All procedures were performed on mice anesthetized with 37.5 mg/kg Zoletil (Virbac) combined with 5 mg/kg xylazine hydrochloride (MilliporeSigma). The body temperature was maintained throughout surgery and imaging by placing mice on a heating pad at 37°C. The mice were injected i.v. with 10 μL Qtracker 705 vascular tracker (Invitrogen, Thermo Fisher Scientific) or 30 μL 1% FITC-Dextran 70 (TdB Labs) to visualize vasculature and 7 μL APC-Ly6G (BioLegend) antibody to label neutrophils 30 minutes before imaging. For visualization of transferred WT or *Piezo1*-cKO neutrophils, mice were injected with fluorescence-labeled antibodies 1 hour before imaging.

#### Lung.

The mice were taped to a custom microscope stage, and a small tracheal cannula was inserted and fixed with a suture and attached to a mouse ventilator (RWD Life Science). After cannulation, the mice were placed in the right lateral decubitus position, and a small surgical incision was made to expose the rib cage. A second incision was made by removing 3 ribs to expose the left lung lobe. A flanged 3D-printing thoracal window with a coverslip was placed above the exposed lung surface, and 20 mmHg suction was applied (Yuyan Instruments) to gently immobilize the lung.

#### Spleen and kidney.

To expose the spleen and liver, mice were placed in a right lateral position, and a skin incision was made on the left flank. The same window used for lung imaging was used to facilitate imaging of the spleen and kidney.

#### Liver.

A midline incision was performed, followed by a lateral incision along the costal margin to the midaxillary line to expose the liver. Mice were placed in a right lateral position, and the ligaments that connect the liver to the diaphragm and the stomach were cut, allowing the liver to externalize onto a glass coverslip. Exposed abdominal tissues were covered with saline-soaked gauze, and the liver was draped with saline-soaked gauze to prevent dehydration. Finally, the mice were placed on a plate with a coverslip for imaging.

#### Brain and meninge.

The skin on the parietal skull bone was surgically removed. The membrane above the skull bone was carefully removed, and a homemade iron imaging plate was glued to the skull bone using dental cement. The skull bone over the barrel cortex was thinned using an electrical micro-drill (RWD, 800-003777-00) to a thickness of approximately 30–40 μm. Second-harmonic signals (SHGs) were used to identify meninge, and the shape and size of vasculature were used to discriminate pia mater from the parenchyma.

Time-lapse images were acquired with an upright microscope (Olympus FluoView FVMPE-RS equipped with an XLPlan N ×25/1.05 objective) for lung, spleen, kidney, brain, and meninge imaging or an inverted microscope (Olympus FluoView FV3000 equipped with a UplanSApo ×20/0.75 objective) for liver imaging. Acquisitions were performed at the following excitation wavelengths: 920 nm for FITC and tdTomato; 1,150 nm for APC; and 800 nm for SHGs. The images were acquired with the following settings: 640 × 640 pixels every 5 seconds for 10 minutes. To exclude artificial results (autofluorescence and potential tissue damage caused by surgery), a single *z* at least 15 μm below the tissue surface was acquired, and all videos were acquired within 1 hour of surgery for each tissue. For the behavioral landscape, all videos were acquired with a single *z*; for in vivo Ca^2+^ imaging, a full *Z*-stack for 6 μm at a 3 μm interval every 5 seconds was captured to generate a 4D image.

### Intravital Ca^2+^ imaging

*Ly6g^Salsa6f^*, WT *Salsa6f*^,^, and *Piezo1*-cKO-*Salsa6f* mice were used to measure intracellular Ca^2+^ levels in vivo. The G/R ratio was determined to generate additional traces showing single-cell and average changes in cytosolic Ca^2+^ over time. For in vivo Ca^2+^ imaging, we generated a new channel specifically for the tracked cells with the “Coloc” function of Imaris to avoid the influence of lung autofluorescence. Average intensities in the green and red channels were calculated for each tracked neutrophil at each time point and exported as a data matrix. Further analysis and visualization were performed in R. The heatmap was plotted with pheatmap, whereas the scatter plot and correlation maps were generated by corrplot and ggplot2, respectively. Furthermore, Spearman’s rank-order correlation analysis was performed and plotted in R.

### Intravital imaging for bacteria catching and ROS production

The S. pneumoniae strain D39 and D39-GFP were provided by Jingren Zhang (Tsinghua University, Beijing, China). The bacteria were cultured in Todd Hewitt Broth (THB) (Hopebio) containing 0.5% yeast extract (Oxoid) at 37°C with 5% CO2 until they reached a mid-logarithmic growth phase (OD600 of 0.6). D39 was i.v. injected into mice. Image acquisition was performed 30 minutes after injection. Phycoerythrin (PE) anti-Ly6G was i.v. injected to label neutrophils right before image acquisition. To detect ROS production in vivo, WT and Piezo1-cKO neutrophils were isolated from BM and were labeled with CellTracker Orange CMRA Dye (Invitrogen, Thermo Fisher Scientific) and the ROS probe DHR123 (Invitrogen, Thermo Fisher Scientific). WT or Pieao1-cKO neutrophils were i.v. injected into mice that had been injected with bacteria 30 minutes before. Image acquisition was performed 30 minutes after injection.

### Image analysis

Image data were analyzed with FIJI and Bitplane Imaris (9.9.1). Imaris was used to detect cells, make masks, and generate tracks with Labkit and its built-in algorithm. Automated generated tracks by Imaris were reviewed and corrected manually. Subsequently, an area filter was added to filter cells less than 75 μm^2^ in size.

### Behavioral landscape

For the behavioral landscape analysis, 118 parameters calculated by Imaris for each track were exported from Imaris and further selected and analyzed in R (3.6.0) using the Seurat (3.2.1) package. According to a published report (7), the input matrix consists of 38 selected parameters (Supplemental Table 1) of 2,584 tracks (1,296 from the lung, 867 from the spleen, and 421 from the liver). PCA was performed, and cells were clustered on the basis of *k*–nearest neighbor graphs using the Louvain algorithm. Finally, a UMAP was generated to visualize the data in a low-dimensional space and displayed through the Seurat built-in function Dimplot. To identify the parameters differentially scored between the 2 clusters, those that showed at least a 0.25-fold difference (log scale) between the 2 groups were selected. The Seurat built-in functions vlnPlot and Doheatmap were used to visualize each subset and plot the parameter value distribution for each group.

### Intravascular staining for CD45

The same mAb clone was used for both intravascular and ex vivo staining (anti-CD45 mAb, clone 30-F11). PE-Cy7 CD45 antibody dilution (8 mg) was injected i.v. through the retro-orbital vein. PB was collected 7 minutes after injection, followed by thorough lung perfusion and collection. The time from antibody injection to PB and lung harvesting in each mouse was 7 minutes and 15 minutes, respectively.

### Tissue processing and flow cytometry

Liver, lung, kidney, and brain were cut into small pieces (<2 mm^2^) and incubated in 3 mL digestion solution (0.5 mg/mL collagenase IV and 50 units/mL DNaseI) for 40 minutes at 37°C with 5% CO_2_. To obtain a single-cell suspension, digested tissues were disrupted by mincing through a 70 μm cell strainer, centrifuged, and resuspended with 1 mL FACS buffer (2% FBS with 1 mM EDTA). The brain cell suspension was pipetted with a Pasteur tube 10 times and was separated by a single-layer 37% Percoll gradient. The dura mater was peeled from the top of skull with fine tweezers and cut into small pieces and incubated in 1 mL digestion solution, followed by mincing through a 70 μm cell strainer. The spleen was mechanically dissociated using a Dounce homogenizer (MilliporeSigma). BM was collected from the tibias and femurs. BALF was collected by 3 × 1 mL lung lavages with PBS via a cannula inserted into the trachea. Total PB was collected at a volume of 1 mL per mouse before perfusion via the retro-orbital sinus with an anticoagulant tube containing 7 μL of 5 mM EDTA. RBCs were removed with ACK lysis buffer. Cell suspensions were washed in PBS for all tissues, followed by live/dead staining (Zombie Aqua, BioLegend) at 1:500 dilution for 10 minutes at room temperature. Nonspecific antibody binding to cells was blocked by incubation with an anti-CD16/32 antibody at 4°C for 15 minutes. Cells were stained by antibody cocktails at 4°C for 25 minutes. Cells were analyzed on a BD LSRFortessa X20 (BD Biosciences) or BD FACSCanto II (BD Biosciences). For intracellular staining, a Foxp3 Transcription Factor Staining Buffer Set (eBioscience) was used to fix and permeabilize cells followed by intracellular staining with antibodies against VEGFA (BioLegend, 512901), IL-1b (BioLegend, 503501), IL-6 (BioLegend, 504501), and Ki67 (BioLegend, 151211) for 1 hour at 4°C prior to staining and flow cytometric analysis. Flow data were further analyzed in FlowJo (10.8.1).

### Neutrophil in vitro stimulation

Mouse neutrophils were isolated from the BM using the Neutrophil Isolation Kit (Miltenyi Biotec) according to the manufacturer’s protocols. Human neutrophils were isolated from PB using the EasySep Direct Human Isolation Kit (STEMCELL Technologies) according to the manufacturer’s protocols. Cells with greater than 95% purity as determined by flow cytometry were used for further experiments. Isolated cells were stimulated with 10 mM Yoda1 in medium containing 30 nm prostaglandin E2 (PGE2). For pulse treatment, neutrophils were precultured for 2 hours to sediment. Following that, half of the volume of the culture medium was gently removed immediately before adding the corresponding volume medium with Yoda1, to reach a final concentration of 10 μM. The pulse treatment was continued for 5 cycles with Yoda1 (10 minutes) and normal medium (10 minutes). Cells were collected for RNA extraction or Western blot analysis at the indicated time points.

### ROS and phagocytosis assay

Neutrophils were sorted from PB, BM, and lung. To avoid activation during digestion, lung tissues were processed as mentioned above without enzyme incubation. For phagocytosis, 5 × 10^3^ neutrophils were incubated with 5 μg/mL Green *E. coli* particles (Invitrogen, Thermo Fisher Scientific) in a 96-well plate for 30 minutes at 37°C. For ROS production, 5 × 10^3^ neutrophils were incubated with 1.25 nM dihydrorhodamine 123 (Invitrogen, Thermo Fisher Scientific) and 10 nM PMA (MilliporeSigma) in a 96-well plate for 30 minutes at 37°C. Fluorescence of the samples was measured by flow cytometry.

### Fabrication of the microfluidic microcirculation mimetic

To mimic cell trafficking in pulmonary vasculatures, a microfluidic microcirculation mimetic was developed. A Cr photomask of the designs was printed commercially (Maxmicro Co.). Using the photomask, the master molds were fabricated at the Soft Material Lab of Maxmicro Co. following standard photolithographic techniques. Briefly, Max 8 2010 Negative photoresist was deposited on silicone wafer substrates using a spin-coater, prebaked, patterned using the masks and a SUSS MA-6 mask aligner, postexposure baked, and finally developed. To increase durability, the master molds were hard-baked at 150°C for 1 minute, after which they were treated with a vapor of tridecafluoro-1,1,2,2-tetrahydrooctyl-1-trichlorosilane (Tokyo Chemical Industry) in a vacuum desiccator overnight, washed with ethanol, and dried with nitrogen perfusion. For soft lithography, polydimethylsiloxane (PDMS) (Sylgard 184, Dow Corning) was combined in a 10:1 w/w ratio of base to curing agent by vigorous mixing and then poured into the master molds, degassed under vacuum for 30 minutes, and baked for 60 minutes at 80°C. After demolding the cured PDMS and cutting out the device, inlet and outlet holes were punched (Maxmicro, 1.5 mm punch). The PDMS devices were cleaned with tape and bonded to glass coverslips using oxygen plasma (PVA TePla IoN40). For the microfluid experiment, BM neutrophils were labeled with CellTracker Green CMFDA Dye (CM-FDA) (Invitrogen, Thermo Fisher Scientific) and were suspended at 5 × 10^5^ cells/mL and pumped into the microfluidic channel by an infusion pump (Pump 11 Elite, Harvard Apparatus) at a speed of 5 mL/minute. The cells that passed through the channels were collected for RNA extraction.

### Transwell

To mimic neutrophil migration through single constriction, a Transwell with a 5 mm pore diameter was used. BM-derived neutrophils were suspended at 5 × 10^5^ cells/mL. A 300 mL cell suspension was added on top of the hanging cell insert and allowed to spontaneously migrate for 30 minutes. Cells were collected from both the insert and the lower well of the plate for further qPCR analysis.

### Western blotting

Neutrophils were treated as indicated and lysed with ice-cold 1× SDS Protein Loading Buffer (Sharebio) containing the 1× Halt Protease and Phosphatase Inhibitor Cocktail (Invitrogen, Thermo Fisher Scientific) with gentle pipetting. Protein lysates were resolved on FuturePAGE 4%–20% Bis-Tris Protein Gels (ACE) and transferred to PVDF membranes (MilliporeSigma). Membranes were incubated with primary antibodies and then HRP-conjugated secondary antibodies (Beyotime) and were detected using the LumiBest ECL substrate solution kit (Sharebio) and the ChemiDoc MP Imaging System (Bio-Rad).

### Adoptive transfer

Neutrophils were isolated from the BM using the Neutrophil Isolation Kit (Miltenyi Biotec). Neutrophils (8 × 10^6^) were transferred into recipient mice by tail-vein injection. Tissues were harvested 2 hours after transfer and then processed for cell sorting. Donor neutrophils were discriminated from recipient neutrophils on the basis of tdTomato expression by flow cytometry. For each tissue, 2 × 10^3^ neutrophils were sorted to lysis/binding buffer (Invitrogen, Thermo Fisher Scientific) for further RNA extraction and qPCR.

### RNA extraction and qPCR

RNA extraction was performed using the Dynabeads mRNA DIRECT Purification Kit (Invitrogen, Thermo Fisher Scientific) following the manufacturer’s instructions. cDNA was generated with PrimeScript RT Master Mix (Takara) according to the manufacturer’s protocols. Reverse transcription qPCR was performed using AceQ Universal SYBR qPCR Master Mix (Vazyme) in 384-well plates and conducted with the QuantStudio 6 Flex Real-Time PCR System (Applied Biosystems). The PCR protocol started with 1 cycle at 95°C (5 minutes) and continued with 40 cycles at 95°C (10 seconds) and 60°C (30 minutes). The 2^–Ct^ method was used to calculate the relative gene expression level, with *Actb* as the housekeeping gene. Detailed primer information is listed in Supplemental Table 3.

### Neutrophil isolation

For bulk and scRNA-Seq, cell suspensions from PB and lung were first isolated using a neutrophil negative selection kit (Miltenyi Biotec) according to the manufacturer’s protocol. For bulk RNA-Seq, FACS was applied to all samples (BM, lung, PB) using the BD FACSAria III (BD Biosciences), and CD45^+^CD11b^+^Ly6G^+^ cells were sorted for sequencing. For human samples, neutrophils were identified as CD45^+^CD11b^+^CD66b^+^ cells. Neutrophils were at a purity of greater than 99% for bulk sequencing and a purity of 90% for single-cell sequencing.

### Neutrophil depletion

For neutrophil depletion, 100 μg anti–mouse Ly6G antibody (Bio X Cell) was i.p. injected at 24 hours or twice at 24 hours and 48 hours prior to analysis, each time resulting in a greater than 85% reduction in circulating neutrophil counts compared with isotype controls (Bio X Cell). In this condition, neutrophils were gated by CD45^+^CD11b^+^Ly6C^int^ cells. For neutrophil depletion in *Ly6g-DTR* mice, 3-week-old mice were i.p. injected with diphtheria toxin (List Biological, 20 ng/g).

### RNA isolation and library preparation for bulk RNA-Seq

Total RNA was extracted using TRIzol reagent (Invitrogen, Thermo Fisher Scientific) according to the manufacturer’s protocol. RNA purity and quantification were evaluated using the NanoDrop 2000 spectrophotometer (Thermo Fisher Scientific). The RNA integrity number (RIN) was evaluated using the Agilent 2100 Bioanalyzer (Agilent Technologies). Only samples with a RIN of greater than 7 were adopted to prepare the library. The libraries were constructed using the VAHTS Universal V6 RNA-Seq Library Prep Kit according to the manufacturer’s instructions. Transcriptome sequencing and analysis were conducted by OE Biotech Co.

### Bulk RNA-Seq process and analysis

The libraries were sequenced on an Illumina NovaSeq 6000 platform, generating 150 bp paired-end reads. Approximately 48,000,000 raw reads were generated for each sample. Raw reads of the fastq format were first processed using fastp, and the low-quality reads were removed to obtain clean reads. Approximately 45,000,000 clean reads for each sample were retained for subsequent analyses. The clean reads were mapped to the reference genome using HISAT2. The fragments per kilobase of transcript per million mapped reads (FPKM) of each gene was calculated, and read counts for each gene were obtained by HTSeq count. PCA analysis was performed using R (version 3.6.0) to evaluate the biological duplication of samples. Differential expression analysis was performed using DESeq2. A *Q* value of less than 0.05 and a log_2_(fold change) of greater than 1 was set as the threshold for significantly DEGs. GO analysis was performed according to DEGs using the R package clusterProfiler.

### Single-cell library construction and sequencing

The scRNA-Seq libraries were generated using the 10X Genomics Chromium Controller Instrument and Chromium Single Cell 3′ V3.1 Reagent Kits (10X Genomics). Briefly, cells were concentrated to approximately 1,000 cells/μL and loaded into each channel to generate single-cell gel beads in emulsions (GEMs). After the RT step, the GEMs were broken, and barcoded cDNA was purified and amplified. The amplified barcoded cDNA was fragmented, A-tailed, and ligated with adapters, and the index PCR was amplified. The final libraries were quantified using the Qubit High Sensitivity DNA assay (Thermo Fisher Scientific), and the size distribution of the libraries was determined using a High Sensitivity DNA chip on a Bioanalyzer 2200 (Agilent Technologies). All libraries were sequenced by the Illumina sequencer (Illumina) on a 150 bp paired-end run.

### scRNA-Seq data processing and filtering

The quality of sequencing reads was evaluated using FastQC and MultiQC. Cell Ranger version 2.2.0 was used to align the sequencing reads (fastq) with the mouse mm10 mouse transcriptome and quantify the expression of transcripts in each cell. This pipeline resulted in a gene expression matrix for each sample, which recorded the number of uniqe molecular identifiers (UMIs) for each gene associated with each cell barcode. The cellular specimens obtained from 2 distinct individuals were combined and subjected to combined analysis. Following the exclusion of low-quality cells and identification of neutrophils, a total of 20,941 cells were obtained, with 9,118 cells originating from PB and 11,823 cells from lung tissue.

### Data integration

The single-cell sequencing data from lung and PB neutrophils were integrated using Canonical Correlation Analysis and the standard workflow from Seurat developers (https://satijalab.org/seurat/v3.2/integration.html). Integrated data were only used for PCA, and all steps relied on PCA (clustering and UMAP visualization). All other analyses (for example, differential expression analysis) were based on the normalized data without integration. A total of 15 principal components were used to perform clustering and UMAP dimensional reduction. We performed the “FindClusters” function (resolution: 0.3) to cluster cells using the Louvain algorithm based on the same principal components as for the “RunUMAP” function. We used the “FindAllMarkers” function [log fold change × threshold = log(1.4)] based on normalized data to identify DEGs. Differential expression was determined on the basis of the nonparametric Wilcoxon rank-sum test. DEGs with adjusted *P* values of greater than 0.05 were excluded. Subsequently, we identified each neutrophil subcluster according to their DEGs regarding former neutrophil nomination by Xie et al. (11) and further confirmed their identification by correlation analysis.

### Electrophysiology

Whole-cell patch-clamp recordings were performed at room temperature using the Molecular Devices system (Axoclamp 200B, Digidata 1440, pCalmp 10). The membrane voltage was maintained at 60 mV. The pipette solution contained the following: 120 mM KCl, 30 mM NaCl, 1 mM MgCl_2_, 0.5 mM CaCl_2_, 5 mM EGTA, 4 mM Mg-ATP, and 10 mM HEPES, pH 7.4, osmolarity kept at 280–300 mOsm/L. The standard external solution (SS) contained the following: 150 mM NaCl, 5 mM KCl, 1 mM MgCl_2_, 2 mM CaCl_2_, 10 mM glucose, and 10 mM HEPES buffered to various pH values with Tris base or HCl. The osmolarity of SS was kept at 300–330 mOsm/L. Mechanical stimulation was applied to cells using a fire-polished glass pipette with a 3–5 μm tip diameter. Experiments were performed with dissociated single cells.

### Live-cell imaging

Ratiometric imaging using Fluo-8 and CMRA dyes measured Ca^2+^ signals in WT and *Piezo1*^Vav1^ neutrophils. The selected BM neutrophils were suspended with 2 μM CMRA (Invitrogen, Thermo Fisher Scientific, C34551) and 5 μM Fluo-8 (Abcam, 1345980-40-6) in HBSS (Solarbio, H1045) and seeded in a glass-bottomed cell culture plate coated with 0.0001% poly-d-lysine (PDL) (Beyotime, ST508) in a glass-bottomed cell culture dish (NEST, 801002) and cultured at 37°C, 5% CO_2_ for 30 minutes. The culture medium was changed to HBSS with calcium and magnesium before imaging. To stimulate a Piezo1-induced calcium influx, 30 μM Yoda1 (MilliporeSigma, SML1558) was topically added to the imaging field during live-cell imaging. An Olympus FluoView FV3000 microscope equipped with an Olympus UplanSApo ×20/0.75 objective was used. We acquired a time-lapse video to record the calcium influx every 3 seconds for 3 minutes at a single *z*.

### Immunofluorescence and confocal imaging

Lungs were infused with 600 μL UltraPure low-melting-point agarose (Invitrogen, Thermo Fisher Scientific, 16520-050; 1.5% in PBS and prewarmed at 40°C) through the trachea. Tissues were harvested and fixed overnight in 4% paraformaldehyde (PFA) (ServiceBio, G1101) and sequentially dehydrated in 15% and 30% sucrose before embedding in the OCT compound (Sakura Finetek, 4583). The sections were cut into 30 μm pieces on a Leica cryostat and permeabilized with 0.1% Triton X-100 for 2 hours, followed by blocking with 0.1% Fc block and 2% BSA (Kingmorn, KS1090) for 1 hour at room temperature. Antibodies were diluted in 0.2% Tween-20 in PBS, and the sections were stained at 4°C in a dark, humidified chamber. The immunofluorescence antibodies used in this study are listed in Supplemental Table 4. After staining, the slides were mounted with an antifade mounting medium (Yeasen) and examined on an Olympus FluoView FV3000 microscope equipped with a UplanSApo ×20/0.75 objective (Olympus).

### LPS-induced acute lung injury

The mice were anesthetized and fixed in a supine position on a sloped intubating platform. The mouse was intratracheally instilled with 50 mL saline or saline containing LPS (2 mg/kg) to induce lung injury. The mice were euthanized after 10 days, and their lungs were collected for flow cytometric analysis of EC proliferation.

### Pulmonary hypertension model

The mice were exposed to hypoxia (10% O_2_) for 3 weeks in a ventilated chamber. The hypoxic environment was maintained as a mixture of air and nitrogen. The chamber was kept closed and was only opened to supply food and water as well as for cleaning twice a week.

### Measurement of RV pressure

RV pressure was measured using an open-chest invasive method (30). Briefly, the mouse was anesthetized and then connected to a small animal ventilator. The thoracic cavity was exposed, and a pressure transducer catheter was inserted into the right ventricle to measure RV pressure. The waveform was displayed on the monitor (GE Healthcare, B105) to ensure correct positioning and data recording.

### Statistics

For most experiments, comparisons between 2 groups were made using a 2-tailed, unpaired Student’s *t* test. Paired, 2-sided Student’s *t* tests were used to compare gene expression between lung and PB–derived neutrophils. The values in each figure represent the means ± SEM. A 1-way ANOVA with Tukey’s multiple-comparisons test, 2-way ANOVA with Šidák’s multiple-comparisons test, or multiple 2-tailed *t* Student’s tests were used for multiple-group comparisons. A *P* value of less than 0.05 was considered statistically significant. All experiments were repeated at least 3 times unless stated otherwise. All statistical analyses and graphics were made using GraphPad Prism (GraphPad Software) and R (The R Project for Statistical Computing). The *n* values in the figure legends indicate the number of biologically independent replicates. The results of Western blotting and immunofluorescent staining are representative of at least 3 biologically independent replicates. Unless otherwise specified, all outcomes were replicated independently more than 3 separate times, producing similar results.

### Study approval

This study complies with all relevant ethical regulations and was approved by the Ruijin Hospital Ethics Committee, Shanghai Jiao Tong University, School of Medicine (2021-376).

### Data availability

All sequencing data generated in this study have been deposited in the NCBI’s Gene Expression Omnibus (GEO) (GSE297134) repository. The published data used in this study were retrieved from the GEO (accession numbers GSE137540). Values for all data points in graphs are reported in the Supporting Data Values file.

## Author contributions

Jin Wang performed most of the experiments, analyzed data, and interpreted results. WYZ, HW, WJB, TLX, XQ, BL, and YQY helped with the experiments. DD and HCL provided human samples. AT and PK helped write the manuscript. Jing Wang designed and supervised the study. Jing Wang wrote the manuscript with input from Jin Wang and WYZ.

## Supplementary Material

Supplemental data

Unedited blot and gel images

Supplemental video 1

Supplemental video 2

Supplemental video 3

Supplemental video 4

Supplemental video 5

Supporting data values

## Version 1 06/02/2025

Electronic publication
