# Supplementary material for: PIEZO1 mediates mechanical reprogramming of neutrophils for proangiogenic specialization in the lung
Source: J Clin Invest. 2025 Jun 2;135(11):e183796. doi: 10.1172/JCI183796 (PMC12126238; doi:10.1172/JCI183796)
Supplement: Supplemental data [file jci-135-183796-s184.pdf]

PIEZO1 mediates mechanical reprogramming of neutrophils for pro-angiogenic specialization in the lung

Authors: Jin Wang[1†], Wenying Zhao[1†], Wenjuan Bai[1†], Dong Dong[2], Hui Wang[1], Xin Qi[3], Ajitha Thanabalasuriar[4], Youqiong Ye[1], Tian-le Xu[3], Hecheng Li[2], Paul Kubes[5], Bin Li[1*], Jing Wang[1*]

Affiliations: [1]Shanghai Institute of Immunology, Shanghai Jiao Tong University School of Medicine, Shanghai, China. [2]Department of Thoracic Surgery, Ruijin Hospital, Shanghai Jiao Tong University School of Medicine, Shanghai, China. [3]Department of Anatomy and Physiology, Shanghai Jiao Tong University School of Medicine, Shanghai, China. [4]Department of Pharmacology and Therapeutics, McGill University, Montreal QC, Canada. [5]Snyder Institute for Chronic Diseases, Cumming School of Medicine, University of Calgary, Calgary, Alberta, Canada

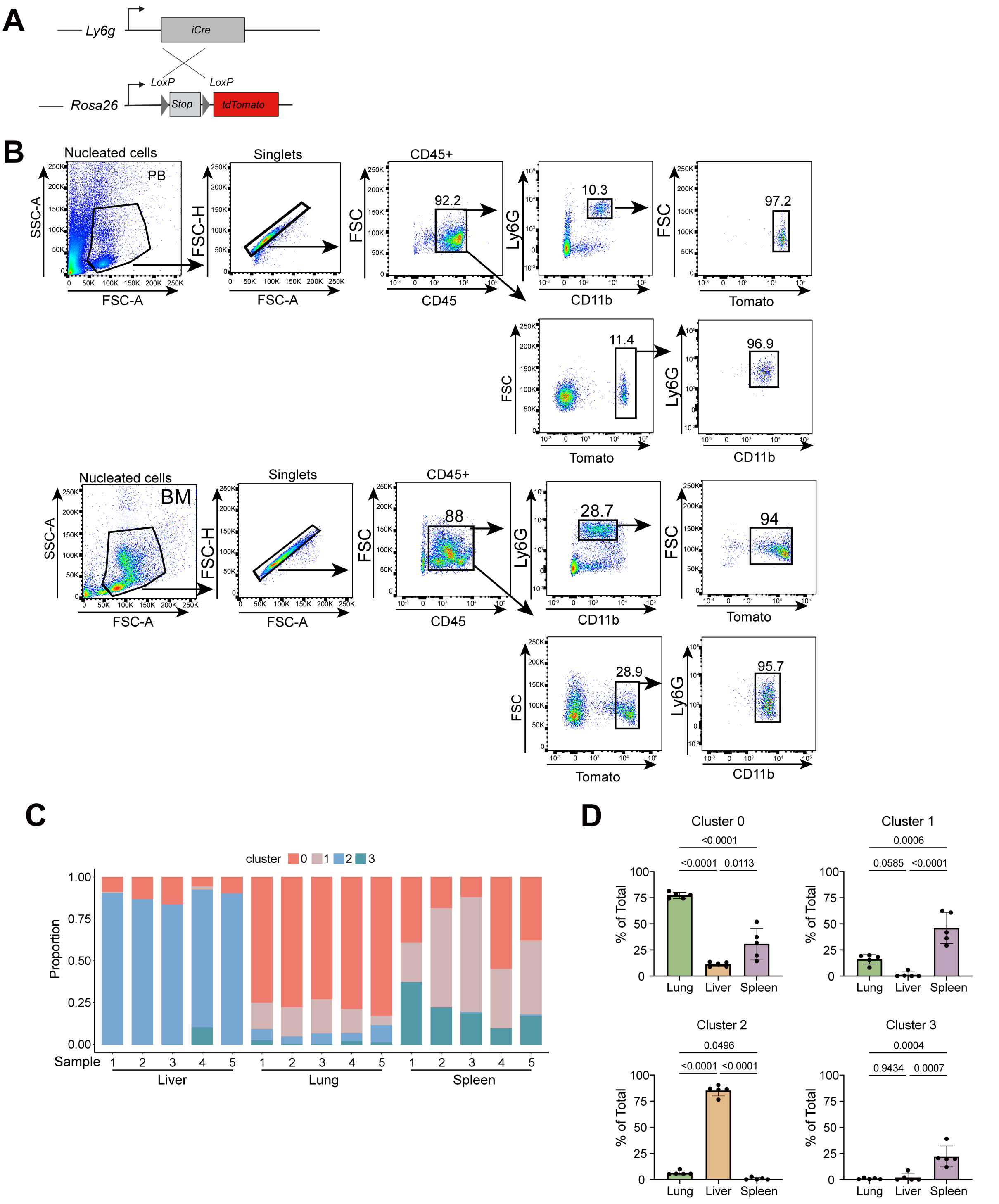

Supplemental Figure 1. Intravital imaging analysis of neutrophil dynamics in different tissues. (A) Construction strategy of *Ly6g-tdTomato* reporter mice. (B) Gating strategy and Flow cytometry analysis of tdTomato expression in PB and BM neutrophils in *Ly6g-tdTomato* reporter mice. (C) Proportions of the four neutrophil clusters in different tissues across individual mouse. (D) Percentage of different neutrophil subpopulations in different tissues. n = 5. Data in D are shown as mean ± s.e.m; one-way ANOVA with Tukey's multiple comparisons test.

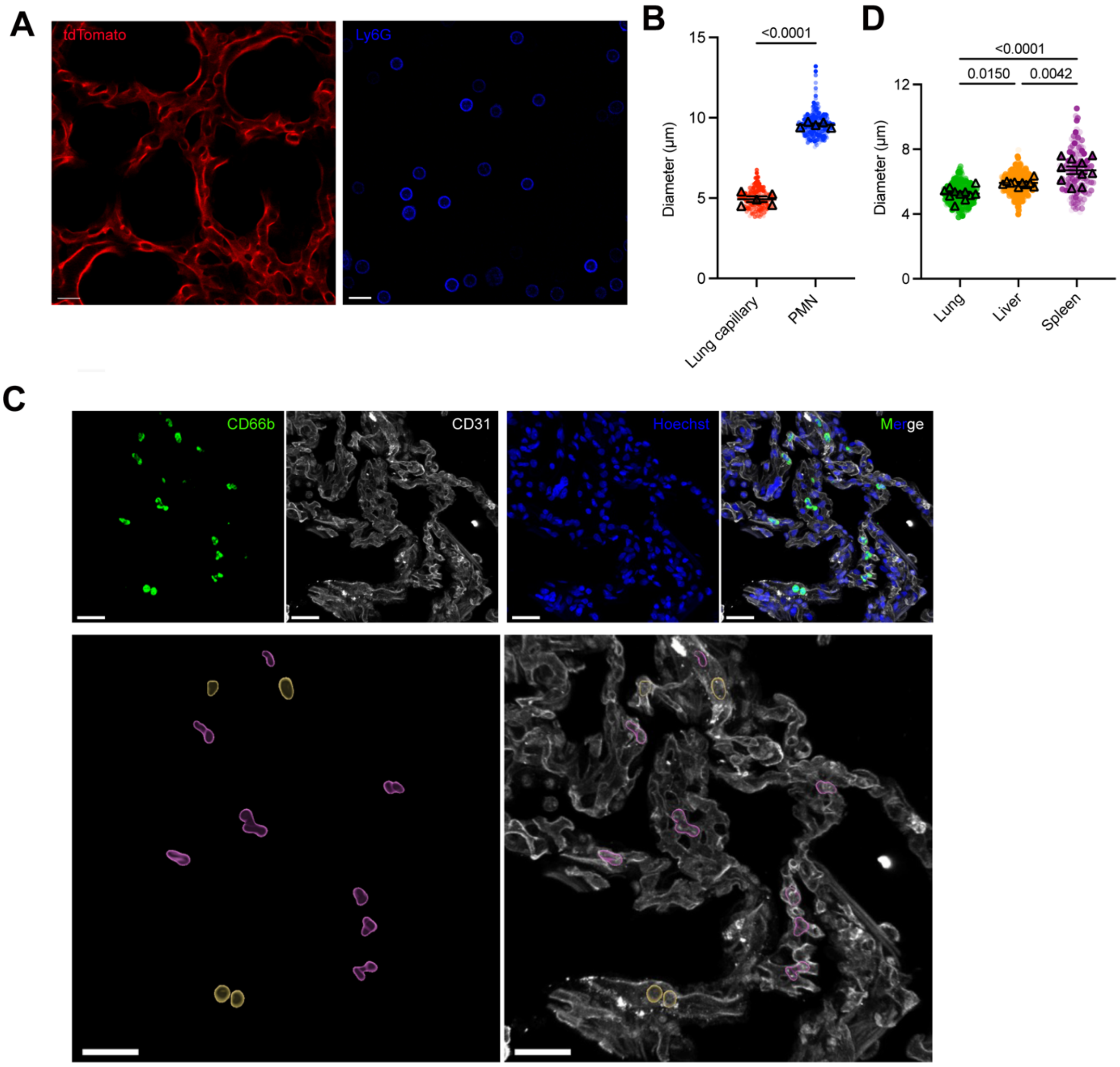

Supplemental Figure 2. Comparison of diameters of neutrophil and pulmonary capillaries. (A) Representative images from intravital imaging of lung vasculature (left panel) and confocal images of neutrophils (right panel). Scale bar, 15 µm. (B) Quantification of diameters of lung capillaries and neutrophils. n = 5. (C) Representative immunostaining of CD31 and the human neutrophil marker CD66b from human lung. Hoechst was used for staining the nucleus. In the lower panel, neutrophils in the capillaries were masked with purple color and neutrophils in the larger vessels were masked with yellow color. Scale bar, 30 µm. (D) Quantification of diameters of blood vessels in lung, liver and spleen. n = 10. Data in B, D are shown as mean ± s.e.m.; one-way ANOVA with Tukey's multiple comparisons (D).

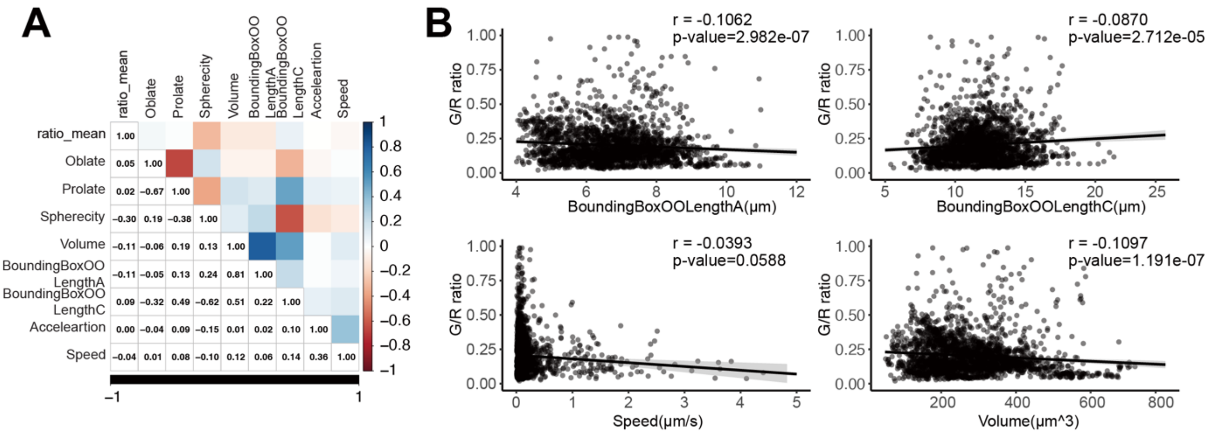

Supplemental Figure 3. Correlation analysis of neutrophil Ca^{2+} with other behavior features. (A) Correlation matrix of paired variables assessed in the cellular behavior and Ca^{2+} analysis from intravital imaging experiments. *P* values are given, and the correlation coefficients are color-coded. (B) Correlation between GCaMP6f/tdTomato intensity ratio (G/R ratio) and indicated parameters describing neutrophil kinetics and morphology traits in vivo. Spearman's rank correlation test (B).

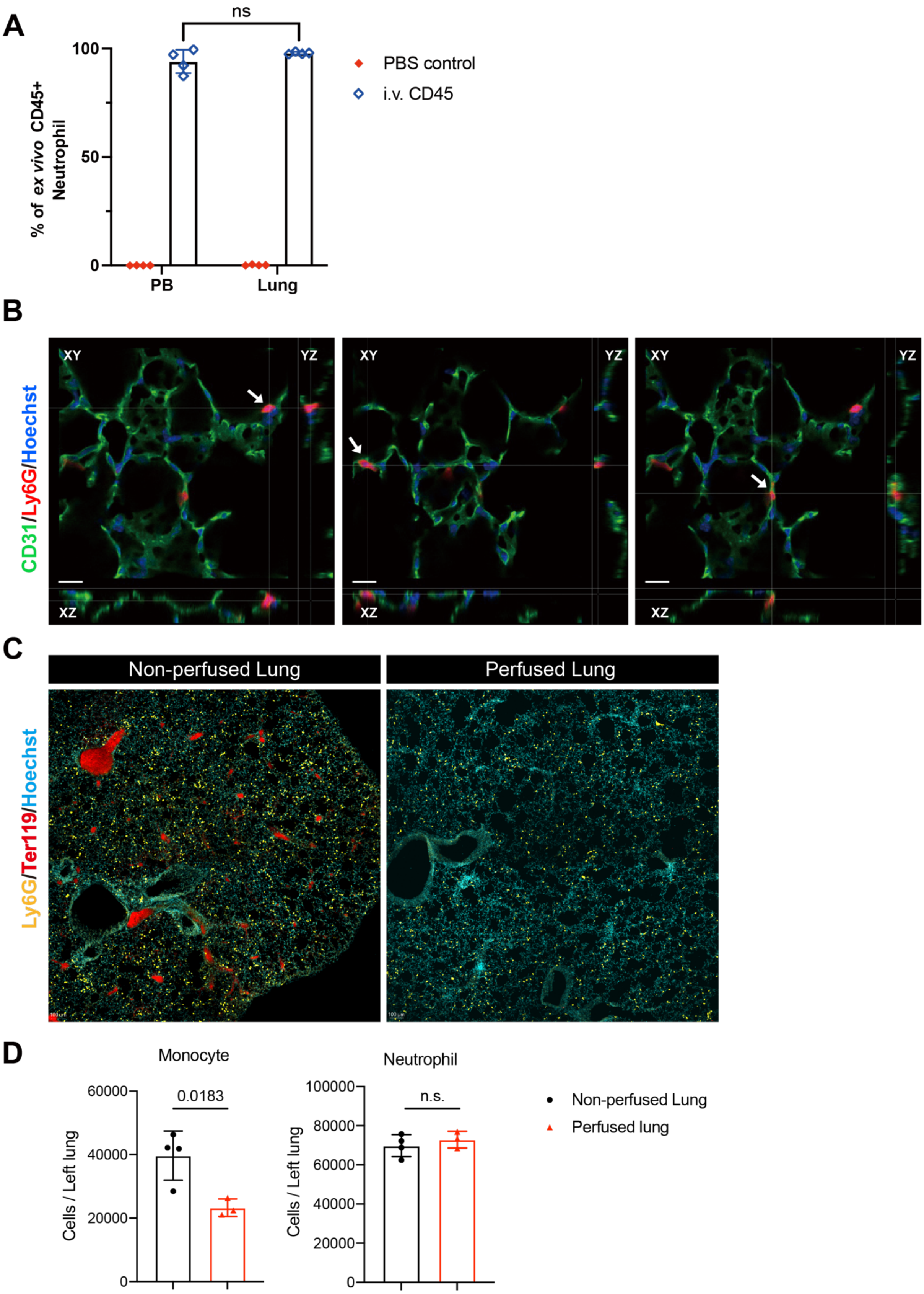

Supplemental Figure 4. Neutrophils are located intravascularly in the lung at steady state. (A) Flow cytometry analysis of cell localization in naïve mice. i.v. CD45 are indicative of cells that are intravascular. n = 4 mice. (B) Representative confocal images of lung

sections showing the relationship between neutrophils and the vasculatures. Scale bar, 15 μm. (C) Representative images of lung sections with or without perfusion. Scale bar, 300 μm. (D) Flow cytometry analysis of monocyte and neutrophil numbers in lungs with or without perfusion. n = 3-4 mice. Data in A and D are shown as mean ± s.e.m.; unpaired two-tailed *t*-test.

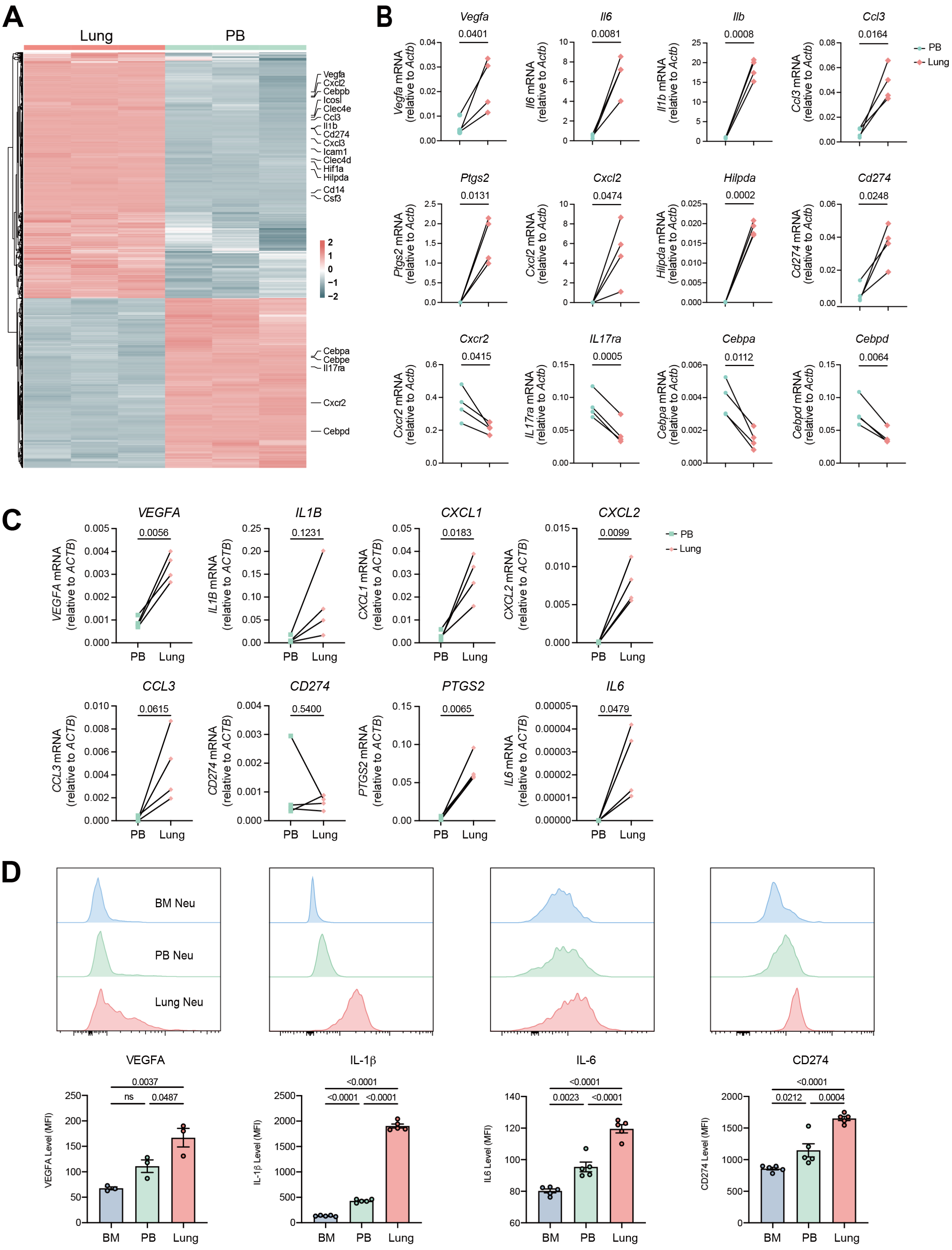

Supplemental Figure 5. Tissue specific signatures in neutrophils. (A) Heatmap of DEGs between lung and PB neutrophils, with several representative genes, indicated at right. (B) mRNA expression of indicated genes in lung and PB neutrophils from the same mouse. n = 4. (C) mRNA expression of indicated genes in neutrophils from human lung tissues and matched peripheral blood. n = 4. (D) Profile of intracellular protein (VEGFA, IL-1β and IL-6) or surface

marker (CD274) in neutrophils from bone marrow, PB and lung neutrophils. n = 3 or 5. Data in D are shown as mean $\pm$ s.e.m.; paired two-tailed *t*-test (B, C); one-way ANOVA with Tukey's multiple comparisons test (D). n.s., not significant.

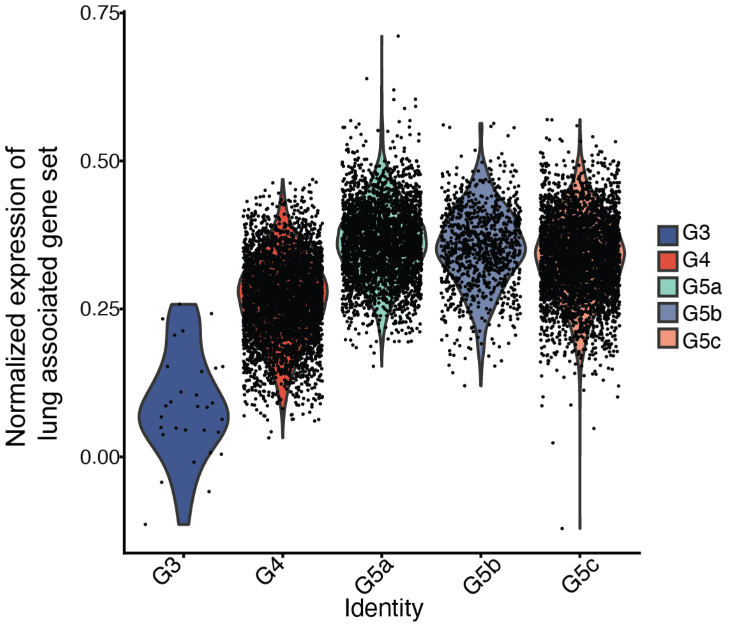

Supplemental Figure 6. Lung specific signature score across neutrophil subpopulations. Lung associated gene set score was calculated and normalized for each neutrophil cluster.

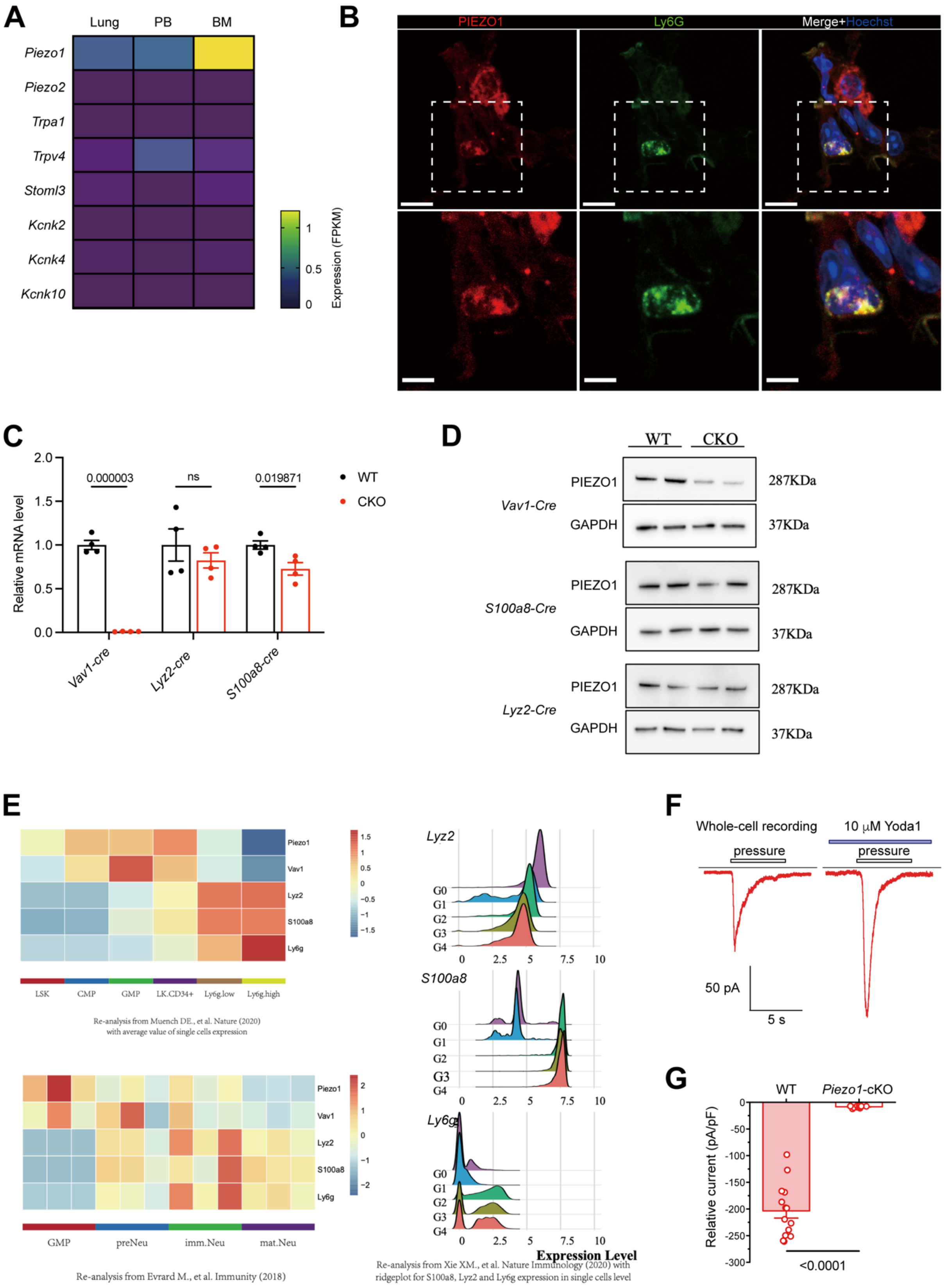

Supplemental Figure 7. Neutrophils in the lung respond to mechanical cues via PIEZO1. (A) Heatmap of different mechanical sensing receptors in neutrophils from different tissues. Data were generated from the log_2FPKM of the mean value of replicates from RNA-seq data.

(B) Representative confocal images of neutrophil staining in lung sections of Piezo1 reporter mice. Scale bar, 10 μm (upper panel), 5 μm (lower panel). (C) mRNA analysis of *Piezo1* expression in purified bone marrow neutrophils from indicated mice. n = 4. (D) Western blot of PIEZO1 in purified bone marrow neutrophils from indicated mice. (E) Comparison of the expression of indicated genes at different stages during neutrophil development. Public data were reanalyzed using R. (F) Representative whole-cell currents of neutrophils in response to poking pressure and adding Yoda1. (G) Whole-cell currents from neutrophils in response to poking pressure. Data in C, G are shown as mean ± s.e.m.; unpaired two-tailed *t*-test.

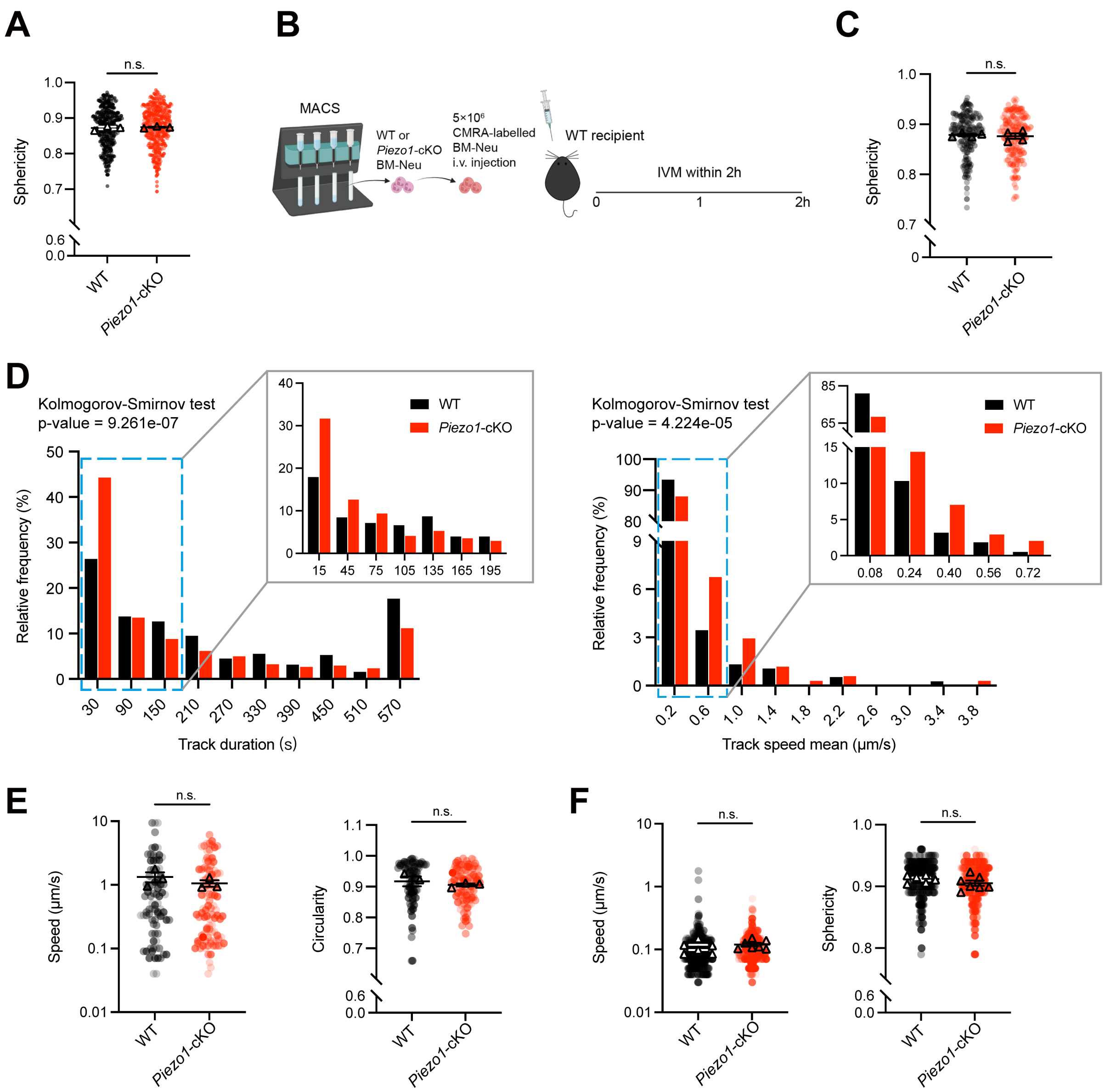

Supplemental Figure 8. Comparison of neutrophil morphology in lung in WT and *Piezo1*-cKO mice. (A) Intravital imaging assessment of neutrophil morphology during their transition in the lung. The degree of cell roundness (sphericity) was quantified using Imaris. n = 3. (B) Schematic of the experimental workflow. (C) Comparison of sphericity between WT and *Piezo1*-cKO neutrophils in the lung. n = 4. (D) Distribution frequency of track duration and average track velocity of WT and *Piezo1*-cKO neutrophils in the lung. (E and F) Intravital imaging analysis of morphological characteristics and dynamics of WT and *Piezo1*-cKO neutrophils in the liver (E) and spleen (F). n = 3 (E). n = 7 (F). Data are shown as mean ± s.e.m.; unpaired two-tailed *t*-test (C, E, F). Two-sample Kolmogorov-Smirnov test (D).

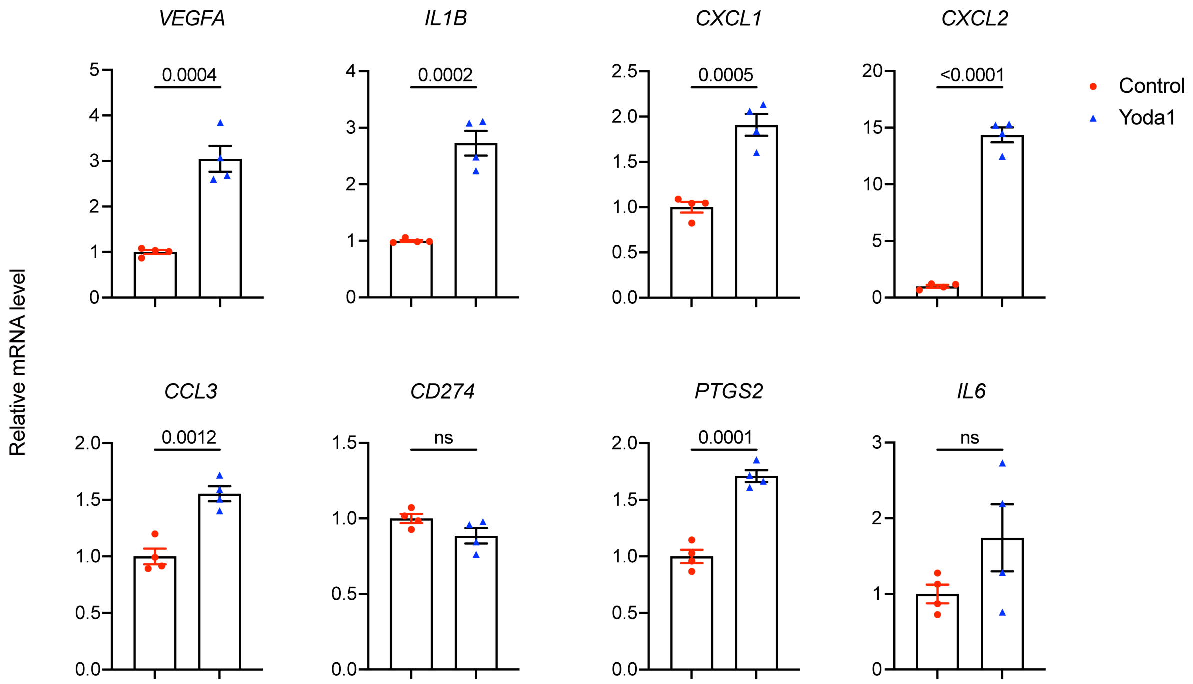

Supplemental Figure 9. Piezo1 activation directly reprograms human neutrophils to express lung associated signatures. Neutrophils from peripheral blood of healthy donors were stimulated with Yoda1 for 2 hours. Relative mRNA expression of the indicated genes in neutrophils was determined. n = 4. Data are mean $\pm$ s.e.m.; unpaired two-tailed *t*-test.

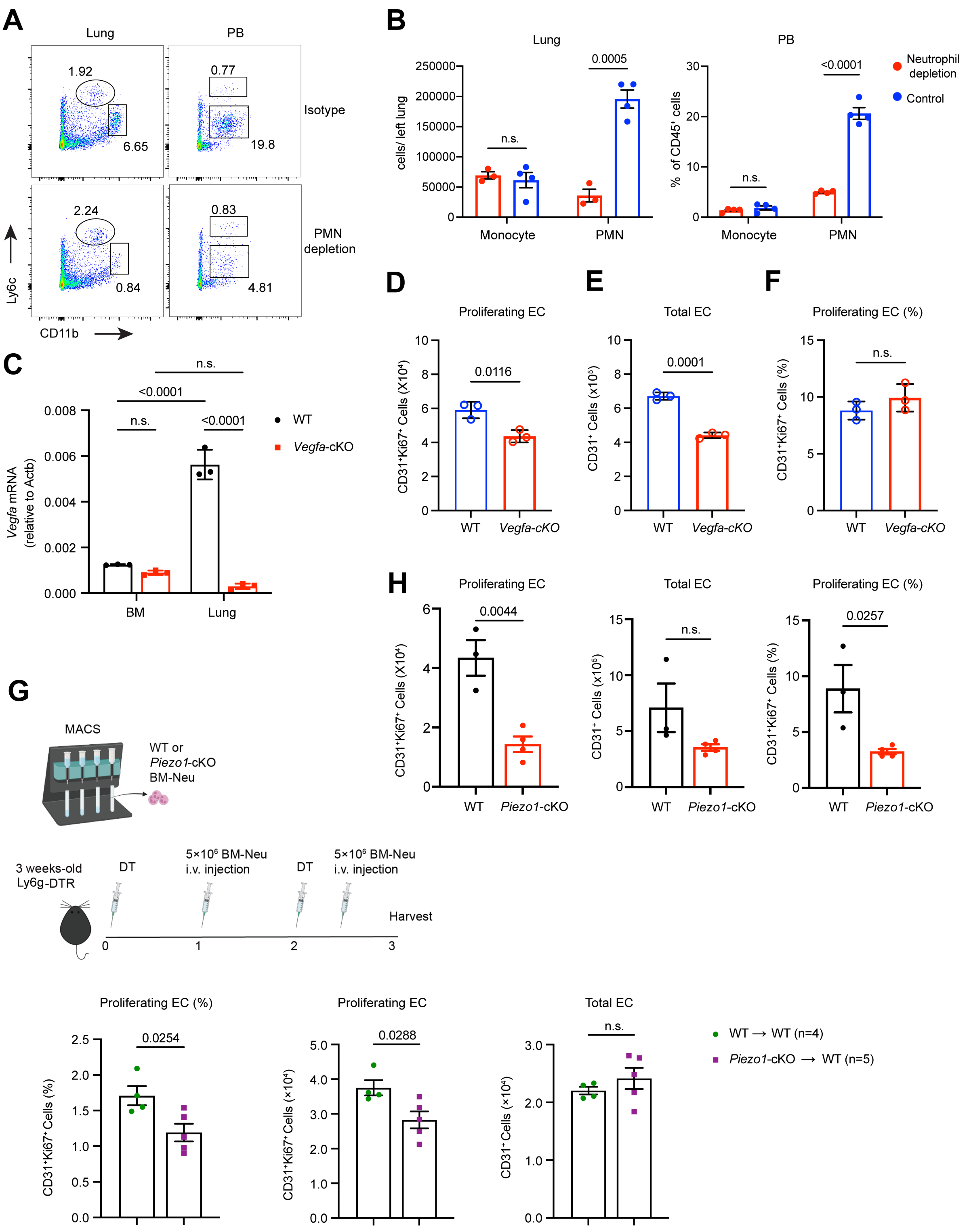

Supplemental Figure 10. Neutrophils sustain angiogenesis via VEGFA. (A) Representative flow cytometry analysis of neutrophils in lung and PB in isotype control (Isotype) and 1A8 antibody (PMN depletion) treated mice. (B) Absolute count (lung) or percentage (PB) of monocyte and neutrophil in isotype control-treated or 1A8 antibody-treated mice. n = 4. (C) mRNA analysis of *Vegfa* expression in indicated tissues from WT and *Vegfa*-cKO mice. n = 3.

(D and E) Flow cytometry assessment of number of $CD31^+Ki67^+$ and $CD31^+$ endothelial cells in lungs from indicated mice. n = 3. (F) Flow cytometry analysis of percentage of $CD31^+Ki67^+$ endothelial cells in lungs from indicated mice. n = 3. (G) Percentage and absolute count of proliferating endothelial cells, as well as the absolute count of total endothelial cells in the lungs of three-week-old *Ly6g-DTR* mice treated as described in the schematic of the experimental workflow. n = 4 or 5 mice per group. (H) Flow cytometry analysis of the percentage and number of CD31+Ki67+ endothelial cells, along with the total endothelial cell number, in WT and *Piezo1*-cko mice 10 days following LPS-induced lung injury. n = 3 or 4 mice per group. Data in B to H are shown as mean ± s.e.m.; unpaired two-tailed *t*-test (B, D to H); one-way ANOVA with Tukey's multiple comparisons test (C)

Supplemental Table 1. Summary of imaging parameters

**Morphology**				
**Track Area Mean**	Track Area Mean	Area max	Area min	Area std
**Ellipsoid Axis Length B**	Track Ellipsoid Axis Length B Mean			
**Ellipsoid Axis Length A**	Track Ellipsoid Axis Length A Mean			
**Ellipsoid Axis Length C**	Track Ellipsoid Axis Length C Mean	Ellipsoid Axis Length C max	Ellipsoid Axis Length C min	Ellipsoid Axis Length C std
**Ellipticity oblate**	Track Ellipticity Oblate Mean	Ellipticity oblate max	Ellipticity oblate min	Ellipticity oblate std
**BoundingBoxOOLength B**	BoundingBox OO Length B max	BoundingBox OO Length B min	BoundingBox OO Length B std	BoundingBox OO Length B mean
**BoundingBoxOOLength C**	BoundingBox OO Length C min	BoundingBox OO Length C max	BoundingBox OO Length C std	BoundingBox OO Length B mean
**H/L ratio**	H/L ratio mean	H/L ratio max	H/L ratio min	H/L ratio std
**Kinetic**				
**Acceleration**	Acceleration max	Acceleration std		
**Displacement**	Track Displacement Length （Displacement max）	Displacement std		
**Speed**	Track Speed Mean	Speed max	Speed min	Speed std

**Displacement Delta**	Displacement Delta Length max	Displacement Delta Length std		
**Track Duration**	Track Duration			
**Track Straightness**	Track Straightness			

Supplemental Table 2 Patient information

Donor Information					
Donor ID	Gender	Surgery date	cTNM stage	Immunoflouresence	qPCR
#001	Female	2023/11/22	IA	Y	N
#002	Male	2023/11/24	IA	Y	N
#003	Female	2023/11/27	IA	Y	Y
#004	Male	2023/11/28	IA	Y	Y
#005	Male	2023/11/29	IA	Y	Y
#006	Female	2023/12/01	IA	Y	Y

Supplemental Table 3 List of primers used in this study

Primers		
**Mouse**		
Gene	Forward (5'-3')	Reverse (5'-3')
Vegfa	CTGCTGTAACGATGAAGCCCTG	GCTGTAGGAAGCTCATCTCTCC
Il1b	ACCTTCCAGGATGAGGACATGA	CTAATGGGAACGTCACACACCA
Cxcl1	CCCTGAAGCTCCCTTGGTTC	TGTTGTCAGAAGCCAGCGTT
Cxcl2	TGTCAATGCCTGAAGACCCT	AACTTTTTGACCGCCCTTGA
Cd274	GCTCCAAAGGACTTGTACGTG	TGATCTGAAGGGCAGCATTTC
Ccl3	TGCTTCTCCTACAGCCGGAA	TGCCGGTTTCTCTTAGTCAGG
Hilpda	TTCCGTGACTCCCCGAGA	ATGCCCAGCACATAGAGGTT
Il6	TCGTGGAAATGAGAAAAGAGTTG	AGTGCATCATCGTTGTTCATACA
Piezo1	GCCCTCATCAAGTGGCTGTA	AGGTGGTCAGTGTTGATGCC
Actb	GGCTGTATTCCCCTCCATCG	CCAGTTGGTAACAATGCCATGT
**Human**		
Gene	Forward (5'-3')	Reverse (5'-3')
Vegfa	TTGCCTTGCTGCTCTACCTCCA	GATGGCAGTAGCTGCGCTGATA
Il1b	CAGAAGTACCTGAGCTCGCC	CCTGGAAGGAGCACTTCATCT
Cxcl1	AGCTTGCCTCAATCCTGCATCC	TCCTTCAGGAACAGCCACCAGT
Cxcl2	TCAATGTGACGGCAGGGAAAT	TCTGCTCTAACACAGAGGGAAAC
Ccl3	GCTCTCTGCAACCAGTTCTCT	TCGCTTGGTTAGGAAGATGACA
Cd274	TGCCGACTACAAGCGAATTACTG	CTGCTTGTCCAGATGACTTCGG
Ptgs2	GAAAACTGCTCAACACCGGA	GCTTCCCAGCTTTTGTAGCC
Il6	GCCCACCGGGAACGAAAG	CGAAGGCGCTTGTGGAG
Actb	CACCATTGGCAATGAGCGGTTC	AGGTCTTTGCGGATGTCCACGT

Supplemental Table 4 List of antibodies used immunofluorescent staining and intravital imaging

Antibodies for IF and IVM				
**First/fluorophore directly conjugated antibody**				
	Fluorephore	Catolog	Titration	Clone
Ly6g	APC	Biolegend 127614	1：200/ 7-10uL per mouse	1A8
Ly6g	PE	Biolegend 127608	1：200/ 7-10uL per mouse	1A8
CD31	/	Servicebio GB11315	1：300	polyclonal
CD66b	AF647	Biolegend 305109	1：20	G10F5
Vegfa	/	Invitrogen MA5-32038	1：100	SP07-01
rabbit anti RFP	/	abcam ab62341	1：100	polyclonal
**Second antibody**				
anti rabbit AF488		Invitrogen A32731	1:1000	
anti rabbit AF488		Cell Signaling 4421S	1:1000	
anti rabbit AF594		Cell Signaling 8889S	1:1000	

Supplemental movie 1. Intravital imaging of neutrophils in different tissues. Intravital imaging was performed in indicated tissues in Ly6G^{tdTom} mice. Mice were treated intravenously with FITC-dextran to label blood vessels. Representative of five independent mice per organ.

Supplemental movie 2. Neutrophils display Ca^{2+} transient while migrating across the lung. Intravital imaging of lungs in Ly6G^{Salsa6f} mice. Mice were treated intravenously with FITC-dextran (grey) to label blood vessels. Arrows indicate cells that display transient Ca2+. Representative of five independent mice.

Supplemental movie 3. No Ca^{2+} events in neutrophils in the spleen and liver. Intravital imaging of spleen and liver in Ly6G^{Salsa6f} mice. Representative of five independent mice.

Supplemental movie 4. Comparison of pulmonary neutrophils migration behaviors and Ca^{2+} signaling in *Piezo1*$^{fl/fl}$*Salsa6f* (WT) and *Piezo1*$^{\Delta Vav1}$*Salsa6f* (Piezo1-cKO) mice. Intravital imaging of lungs in WT-*Salsa6f* and Piezo1-cKO-*Salsa6f* mice. Mice were treated intravenously with fluorescent anti-Ly6G to label neutrophils. Representative of five independent mice.

Supplemental movie 5. Visualizing neutrophil migration through microfluid device. CMFDA labeled neutrophils were perfused to passing through multiple confinements of the microfluidic system. Representative of five independent mice.
