# Supplementary material for: PIEZO1 mediates mechanical reprogramming of neutrophils for proangiogenic specialization in the lung
Source: J Clin Invest. 2025 Jun 2;135(11):e183796. doi: 10.1172/JCI183796 (PMC12126238; doi:10.1172/JCI183796)

Full gel for Figure 4D

pErk

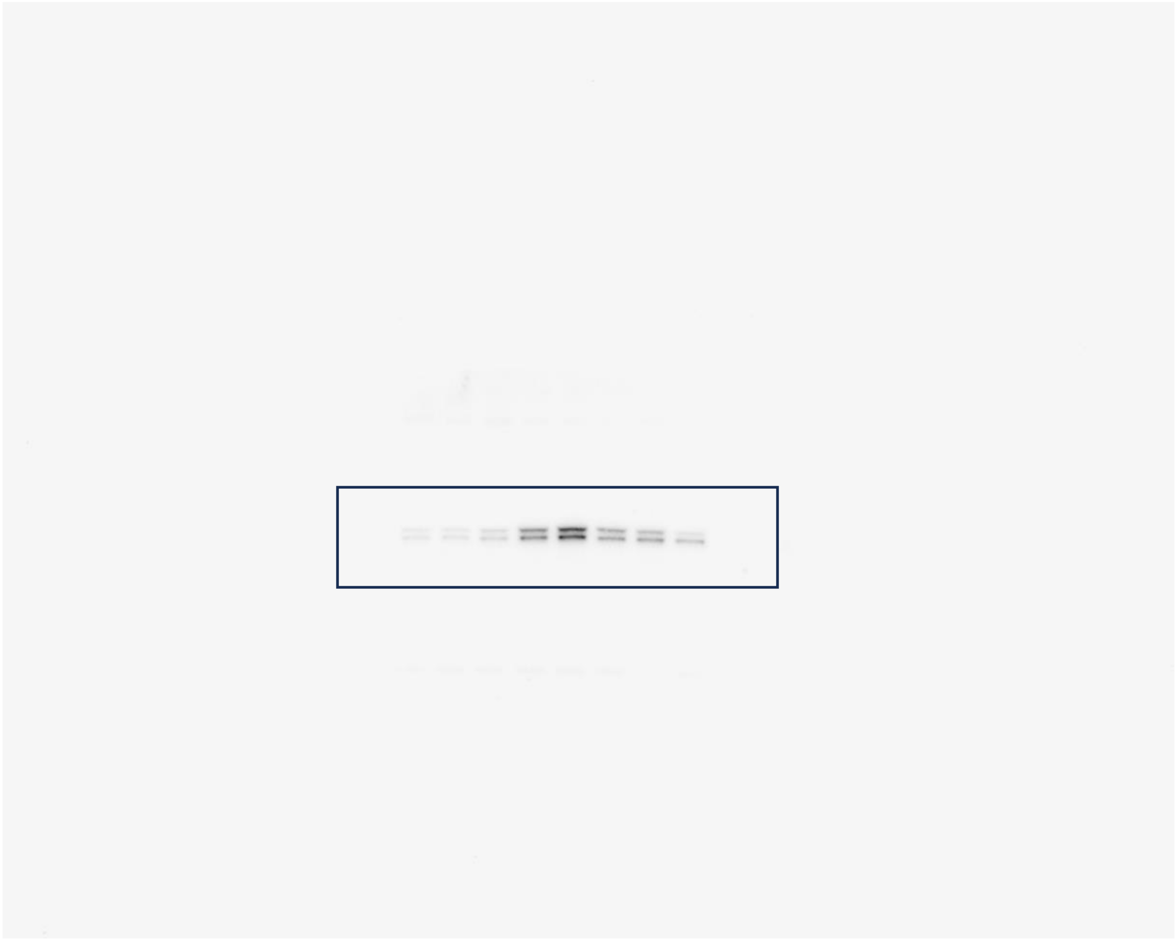

Erk

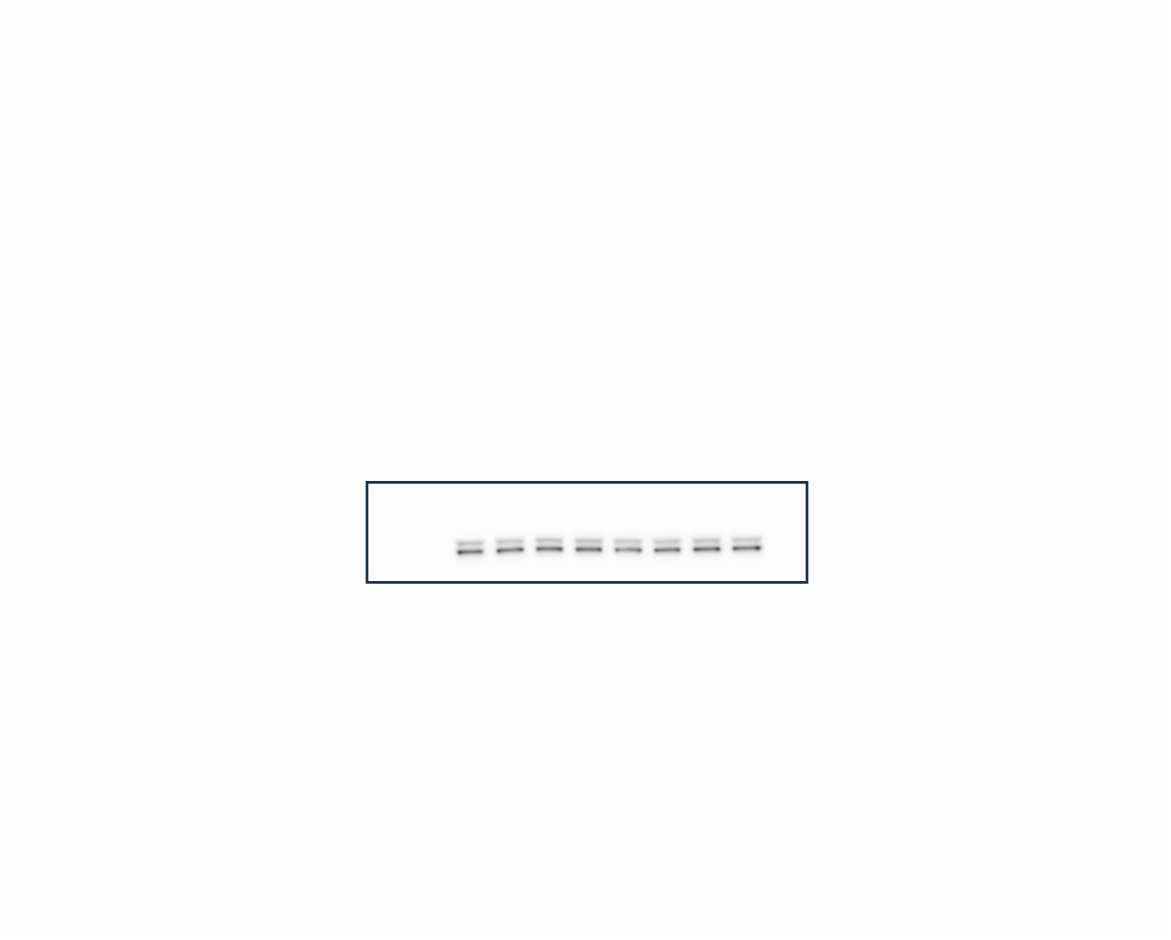

Full gel for Figure 4D

pNF-kB

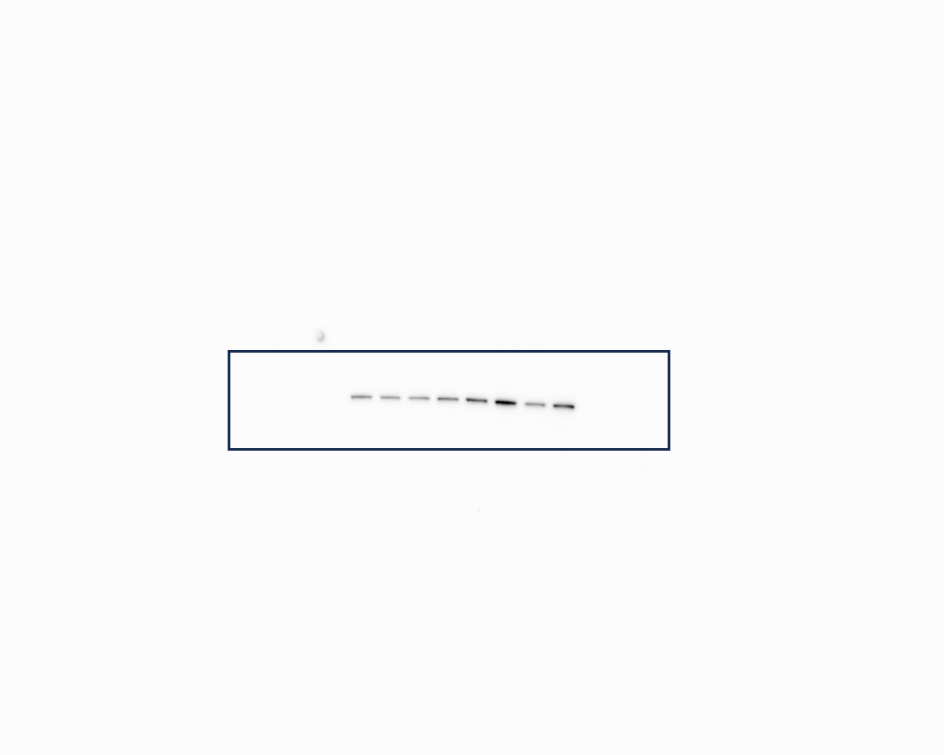

NF-kB

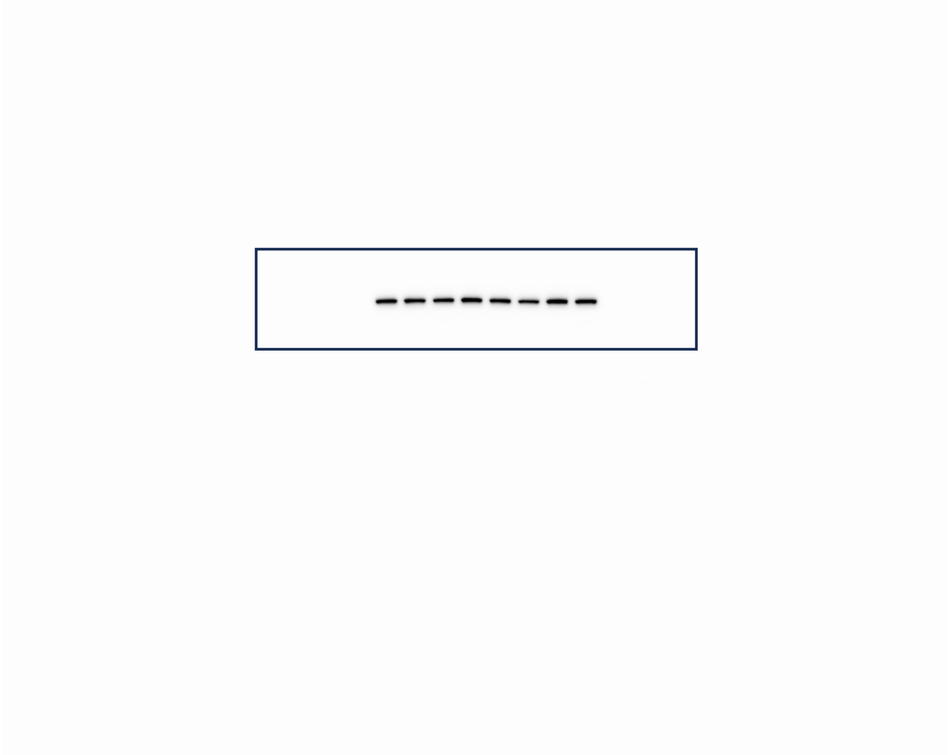

Full gel for Supplemental figure 7D

Vav-cre PIEZO1

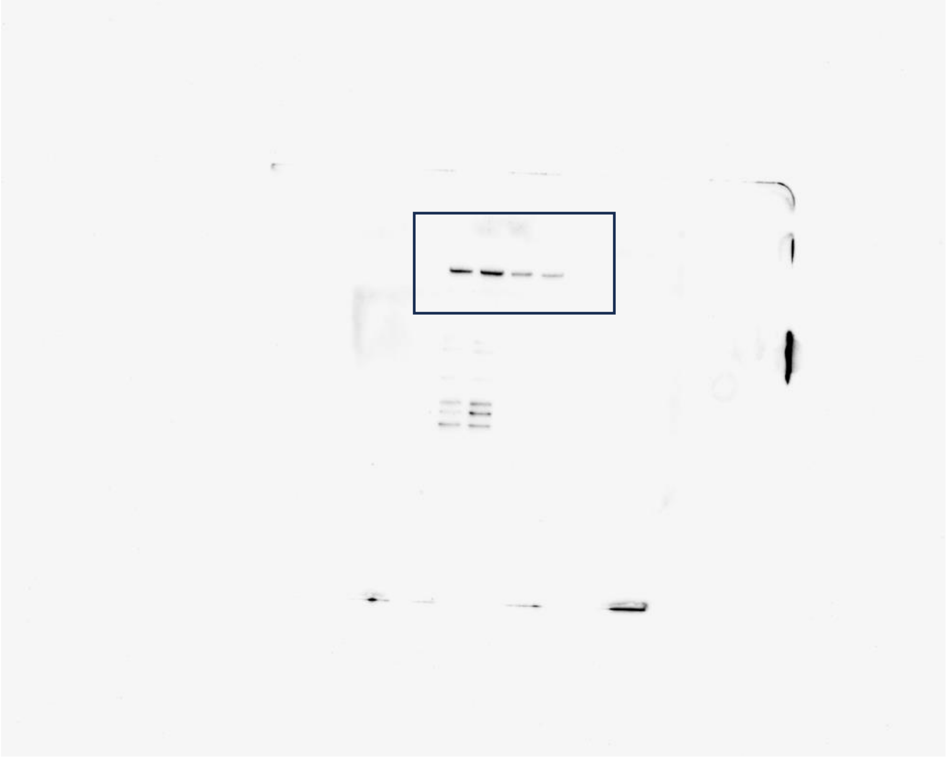

Vav-cre GAPDH

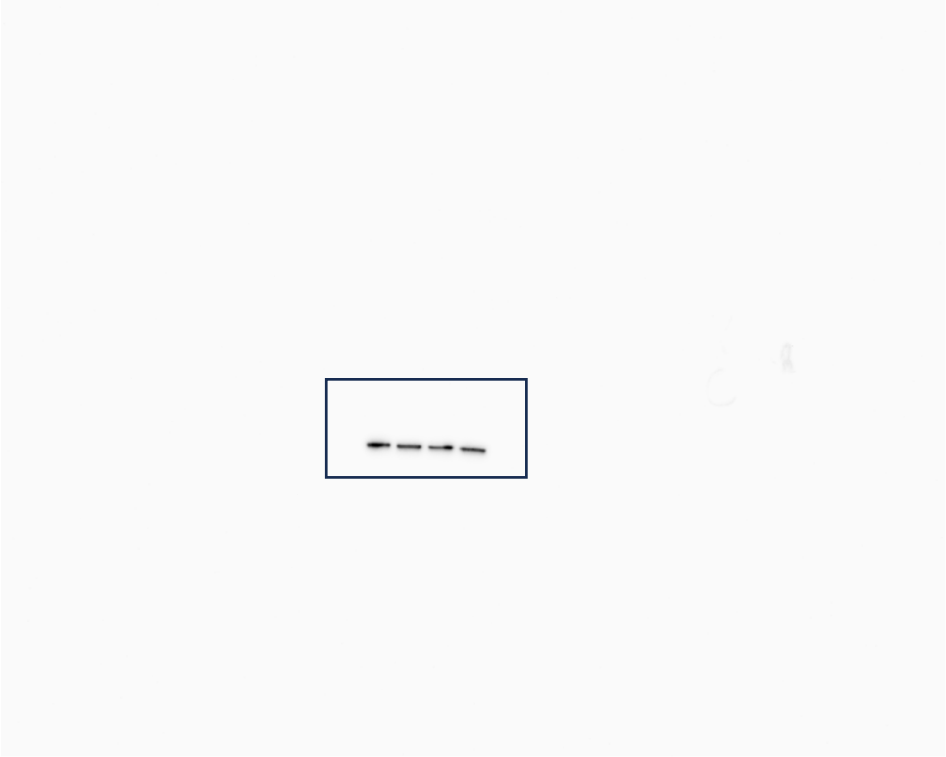

Full gel for Supplemental figure 7D

S100a8-cre PIEZO1

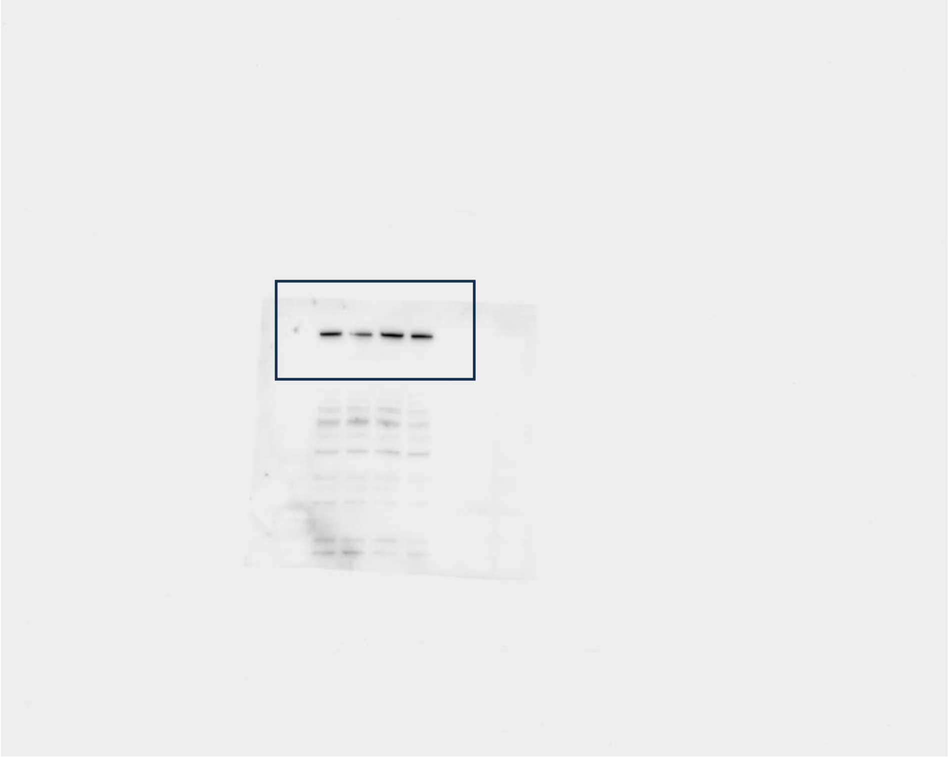

S100a8-cre GAPDH

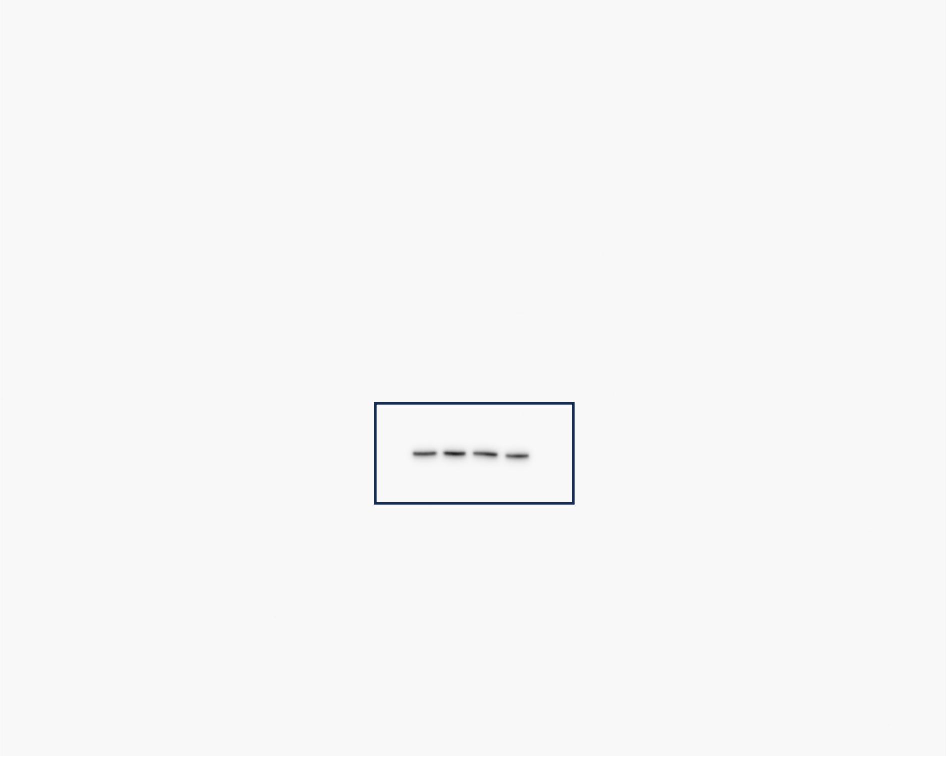

Full gel for Supplemental figure 7D

Lyz2-cre PIEZO1

Lyz2-cre GAPDH

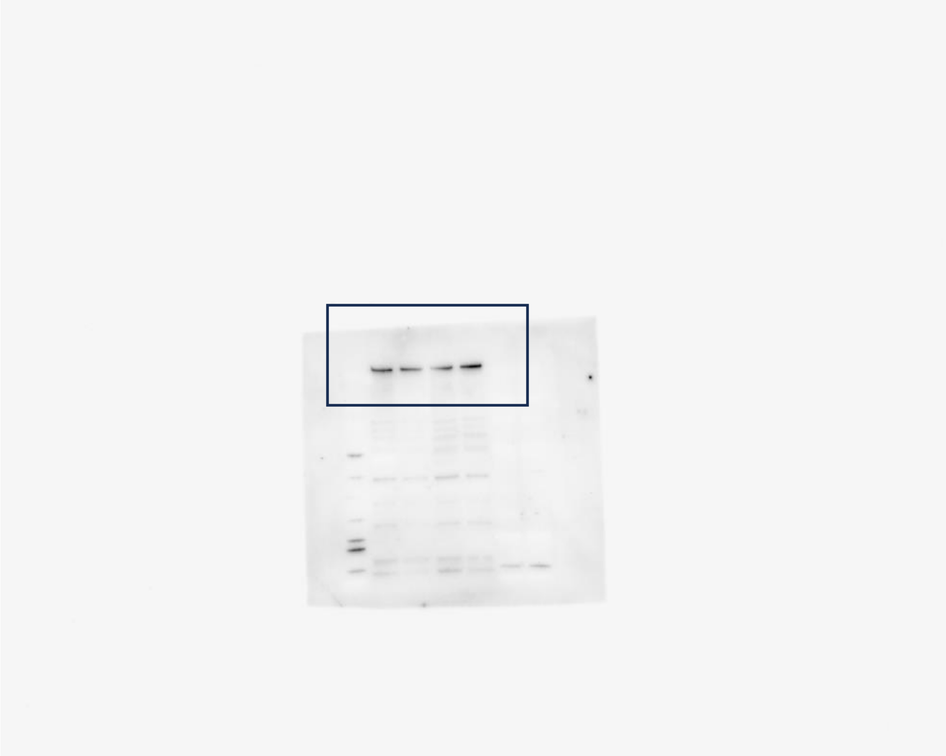

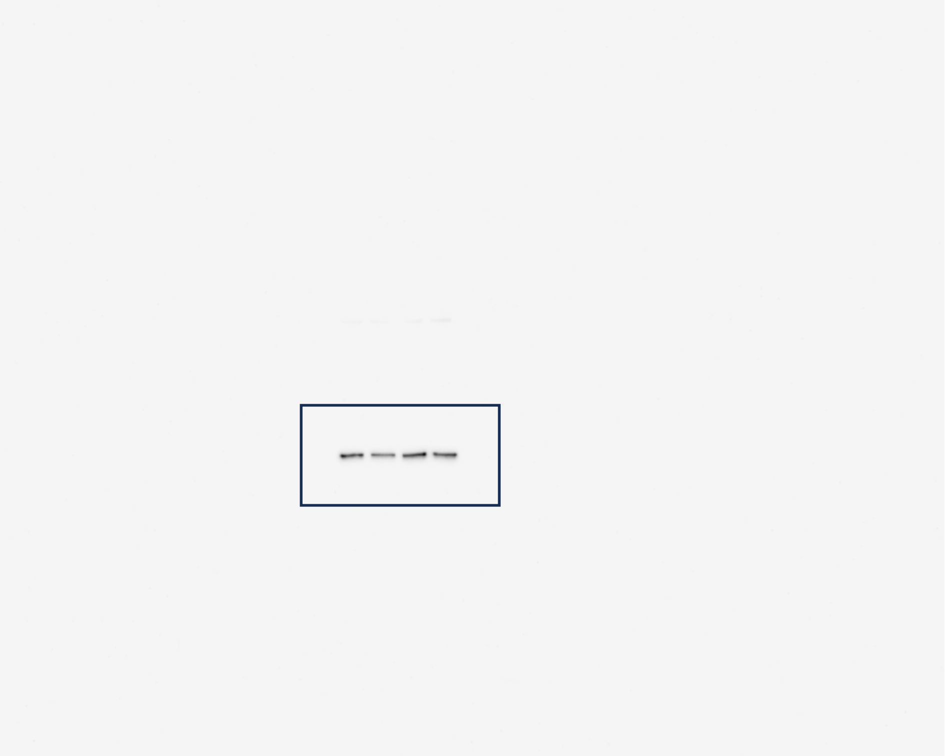

Supplement: Unedited blot and gel images [file jci-135-183796-s185.pdf]
